# Wide‐Bandgap Cu(In, Ga)S_2_ Solar Cell: Mitigation of Composition Segregation in High Ga Films for Better Efficiency

**DOI:** 10.1002/smll.202405221

**Published:** 2025-01-08

**Authors:** Damilola Adeleye, Mohit Sood, Arivazhagan Valluvar Oli, Tobias Törndahl, Adam Hultqvist, Aline Vanderhaegen, Evandro Martin Lanzoni, Yucheng Hu, Gunnar Kusch, Michele Melchiorre, Alex Redinger, Rachel A. Oliver, Susanne Siebentritt

**Affiliations:** ^1^ Department of Physics and Materials Science University of Luxembourg Esch‐sur‐Alzette L‐4365 Luxembourg; ^2^ Department of Materials Science and Engineering Uppsala University Uppsala 75103 Sweden; ^3^ Department of Materials Science and Metallurgy University of Cambridge Cambridge CB3 0FS UK

**Keywords:** (Zn,Sn)O buffer layer, bulk defect, cathodoluminescence, composition segregation, Cu(In, Ga)S_2_, photoluminescence, quasi Fermi level splitting, tandem solar cell

## Abstract

Cu(In, Ga)S_2_ demonstrates potential as a top cell material for tandem solar cells. However, achieving high efficiencies has been impeded by open‐circuit voltage (V_OC_) deficits arising from In‐rich and Ga‐rich composition segregation in the absorber layer. This study presents a significant improvement in the optoelectronic quality of Cu(In, Ga)S_2_ films through the mitigation of composition segregation in three‐stage co‐evaporated films. By elevating the substrate temperature during the first stage, the intermixing of In and Ga is promoted, leading to reduced Cu(In, Ga)S_2_ composition segregation. Furthermore, the optimization of Cu‐excess during the second stage minimizes non‐radiative voltage loss. These combined strategies yield quasi‐Fermi level splitting exceeding 1 eV and a record V_OC_ of 981 mV in Cu(In, Ga)S_2_ devices. Consequently, a champion device achieves an in‐house power conversion efficiency (PCE) of 16.1% (active area) and a certified PCE of 14.8%, highlighting the potential of Cu(In, Ga)S_2_ as a stable and efficient top‐cell device for tandem photovoltaics.

## Graphical abstract

Cu(In, Ga)S_2_ is a stable, cost‐effective alternative top‐cell for tandem solar cells. Optimized growth parameters mitigate compositional segregation, and achieve high bandgap and high optoelectronic quality, while suitable buffer and window layers minimize interface losses. This approach yields a 1.63 eV bandgap solar cell with a record 981 mV open‐circuit voltage and 16.1% power conversion efficiency, advancing Cu(In, Ga)S_2_ top‐cells for tandem applications.

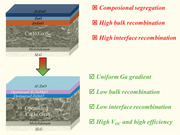

## 1 Introduction

Tandem solar cells have demonstrated their potential to exceed the efficiency limits of single‐junction devices.^[^ ^1^ ^]^ However, current tandem solar cells are fabricated either using costly yet stable III‐V technologies,^[^ ^2^, ^3^ ^]^ or incorporating perovskite as top cell material,^[^ ^4^, ^5^ ^]^ which exhibits lower stability when compared to its bottom cell counterpart silicon.^[^ ^6^ ^]^ Consequently, there is a pressing need for a top‐cell material that is cost‐effective and as stable as current bottom‐cell technologies. Cu(In, Ga)S_2_ has emerged as a promising candidate for top cell applications owing to its tunable bandgap (1.5–2.45 eV),^[^ ^7^, ^8^, ^9^, ^10^, ^11^ ^]^ and the established stability^[^ ^12^, ^13^, ^14^, ^15^ ^]^ of chalcopyrite solar cells. However, despite the remarkable certified power conversion efficiency (PCE) of 23.35% and 23.64% for selenide‐based single‐junction Cu(In, Ga)(S, Se)_2_^[^ ^16^ ^]^ and (Ag, Cu)(In, Ga)Se_2_^[^ ^17^ ^]^ respectively, pure sulfide Cu(In, Ga)S_2_ single‐junction solar cells exhibit a significantly lower certified PCE of only 15.5%.^[^ ^18^ ^]^

Nevertheless, recent advancements in Cu(In, Ga)S_2_ research have yielded significant results in addressing key challenges that cause voltage losses in Cu(In, Ga)S_2_, namely non‐radiative recombination in the bulk and at the absorber interface.^[^ ^19^ ^]^ Notably, absorbers grown under Cu‐deficient conditions at higher temperatures (>570 °C) have reduced density of non‐radiative recombination centers, leading to enhanced optoelectronic performance, specifically higher quasi‐Fermi level splitting (QFLS) and longer minority carrier lifetime.^[^ ^19^, ^20^, ^21^, ^22^, ^23^ ^]^ Furthermore, the utilization of suitable buffer layers like Zn(O, S) and (Zn, Mg)O, has minimized interface recombination.^[^ ^19^, ^24^, ^25^ ^]^ These improvements have resulted in devices with open‐circuit voltages (V_OC_) exceeding 1 V;^[^ ^24^ ^]^ However, in these cells, the fill factor (FF) was low, likely due to barriers in a non‐optimized window. The band alignment on both front interfaces, that is, the absorber/buffer and buffer/i‐layer, is critical. Its optimization requires adaptation of the buffer and the i‐layer.^[^ ^25^ ^]^ Here, we show that the combination of a ZnSnO buffer with a ZnMgO i‐layer can solve the FF issue.

In Cu(In, Ga)(S, Se)_2_ films, the incorporation of Ga plays a crucial role in tuning bandgap and influencing phase stability.^[^ ^11^, ^26^, ^27^, ^28^, ^29^, ^30^, ^31^, ^32^, ^33^ ^]^ Notably, Ga gradients have become a characteristic feature of these films, presenting significant advantages for high‐efficiency devices. These benefits include minimized interface recombination losses and improved charge carrier collection.^[^ ^34^ ^]^ A Ga‐gradient has also been successfully implemented in Cu(In, Ga)S_2_ films through various deposition techniques such as sulfurization of metal precursors^[^ ^18^, ^35^ ^]^ and multistage co‐evaporation processes.^[^ ^19^, ^20^, ^36^ ^]^ While a Ga‐gradient in conjunction with Cu deficiency, achieved by the three‐stage co‐evaporation method, has undeniably enhanced the optoelectronic performance in low‐Ga Cu(In, Ga)S_2_ by reducing front and back interface recombination,^[^ ^19^, ^22^, ^24^ ^]^ replicating similar success becomes more challenging at higher Ga content, particularly for devices targeting bandgaps exceeding 1.6 eV.^[^ ^36^ ^]^

Composition segregation has been identified as an undesirable characteristic in Cu(In, Ga)S_2_ absorbers with higher Ga content.^[^ ^36^, ^37^ ^]^ In such films, X‐ray diffraction (XRD) analysis revealed the presence of two distinct phases within the film; one with a high In concentration (low Ga) with a [Ga]/([Ga]+[In]) (GGI) ratio as low as 0.1, and another with a high Ga concentration (low In) exhibiting a GGI ratio as high as 0.9.^[^ ^36^ ^]^ Such phenomenon has been correlated with the Ga profile in SIMS and GDOES analysis which shows that these two phases form two layers within the film, with the low Ga layer near the surface and the low In layer near the back surface of the absorber.^[^ ^36^ ^]^ This segregation results in an almost abrupt, step‐like downshift in the conduction band minimum toward the front contact, rather than the desired gradual gradient. This phenomenon of composition segregation is attributed to the inherently narrow window of existence for the chalcopyrite Cu(In, Ga)S_2_.^[^ ^27^ ^]^ The phase diagram shows the formation of secondary phases already with small deviations from ideal composition, which are detrimental to device performance.^[^ ^20^, ^22^, ^27^, ^38^ ^]^ Consequently, it was posited that the utilization of the standard three‐stage deposition process,^[^ ^36^ ^]^ which proceeds through extremely Cu‐poor phases, is not suitable for fabricating wide bandgap Cu(In, Ga)S_2_ devices despite this process having been successfully employed in previous record selenide Cu(In, Ga)Se_2_ solar cells.^[^ ^39^ ^]^

To address the composition segregation in Cu(In, Ga)S_2_ films, Barreau et al. proposed a modification of the three‐stage deposition process.^[^ ^22^ ^]^ This approach involves a progressive reduction of the Ga flux while simultaneously increasing the In flux during the first stage, with only In supplied by the end of this stage.^[^ ^22^ ^]^ This modified three‐stage deposition yielded Cu(In, Ga)S_2_ films with smooth Ga‐gradient and facilitated the fabrication of solar cells with a low V_OC_ deficit and enhanced charge transport, which achieved a promising PCE of 16%.^[^ ^22^ ^]^ However, the device bandgap of 1.55 eV remains below the optimal value (≈1.6–1.7 eV) for a top cell in tandem solar cell applications.^[^ ^40^ ^]^

This investigation explores techniques for minimizing composition segregation in Cu(In, Ga)S_2_ absorbers with a bandgap of ≈1.6 eV by systematically varying deposition parameters within the three‐stage process. The main parameters investigated were the substrate temperature during the first stage, since higher growth temperatures are crucial for high‐quality sulfide chalcopyrites,^[^ ^21^, ^26^, ^41^, ^42^, ^43^ ^]^ as well as the Cu‐excess during the second stage. The latter is inspired by studies on the related selenide chalcopyrites which showed that this parameter strongly influences crystallinity and grain size.^[^ ^44^, ^45^, ^46^, ^47^, ^48^ ^]^ This study examines the impact of elemental flux ratios, first‐stage substrate temperature, and Cu‐excess on Ga (and thus bandgap) gradient, particularly on the minimum bandgap (notch), which is a critical region for light absorption and recombination in Cu(In,Ga)S_2_ films. As shown in detail below, a higher first‐stage substrate temperature mitigates composition segregation, which leads to the desired gradual Ga gradient in Cu(In,Ga)S_2_ absorbers. However, this increase in temperature also reduces the Ga content at the back contact (metal surface), thereby promoting high back‐surface recombination. To counteract this effect, surplus Ga was introduced during the first stage of deposition. Moreover, this research demonstrates that a lower Cu‐excess during the second stage after the first stoichiometric point increases the bandgap minimum and minimizes non‐radiative recombination. Solar cells were fabricated on the Cu(In,Ga)S_2_ absorbers to achieve enhanced device performance. To this end, a Zn_1‐x_Sn_x_O_y_ buffer layer and an Al:ZnMgO i‐layer were optimized to reduce interfacial losses. These combined advancements resulted in a device with an active area PCE of 16.1% and a certified PCE of 14.8%, with a record V_OC_ of 981 mV in a Cu(In,Ga)S_2_ device with a bandgap exceeding 1.6 eV.

## 2 Results and Discussion

### 2.1 Influence of Growth Parameters on Cu(In,Ga)S_2_ Optoelectronic Properties

The Cu(In,Ga)S_2_ films investigated in this report were synthesized utilizing the standard three‐stage process,^[^ ^49^ ^]^ similar to the previous report by Shukla et al.,^[^ ^19^ ^]^ and as illustrated in Figure S1 (Supporting Information). As mentioned above, although the modified three‐stage deposition process proposed by Barreau et al. offered smooth composition gradients,^[^ ^22^ ^]^ it produced a device with a bandgap as low as 1.55 eV, which was insufficient for the target bandgap in this study. Therefore, the standard three‐stage deposition process, which has been successfully employed in previous record selenide Cu(In,Ga)Se_2_ solar cells,^[^ ^49^, ^50^ ^]^ was selected. This process provides more precise control over the deposition parameters and film growth profiles, leading to an improved reproducibility of the deposition process for similar films.

Our pre‐study showed that the surface GGI is critical in Cu(In,Ga)S_2_ solar cells.^[^ ^37^ ^]^ A high surface GGI resulted in a conduction band barrier at the absorber‐buffer interface, impeding charge carrier transport, which subsequently affects the FF of the solar cell. To address this issue, we decreased the Ga flux in the third stage compared to the first stage of the Cu(In,Ga)S_2_ deposition process presented in Table S1 (Supporting Information), thereby decreasing the surface GGI and improving charge carrier transport.^[^ ^24^, ^37^ ^]^

### 2.2 Suppressing Composition Segregation Through First‐Stage Deposition Conditions

The slower migration and reaction speed of Ga compared to In, as well as the higher melting point of Ga‐based compounds compared to In‐based compounds,^[^ ^51^, ^52^, ^53^, ^54^, ^55^ ^]^ has necessitated that Ga‐rich Cu(In,Ga)S_2_ be grown at higher substrate temperatures.^[^ ^21^, ^41^, ^56^ ^]^ Furthermore, sulfides exhibit higher melting points than selenides,^[^ ^57^, ^58^, ^59^ ^]^ thus, the synthesis of sulfide absorbers requires higher substrate temperature than their selenide counterparts.^[^ ^20^, ^41^, ^60^ ^]^ In various previous studies, higher substrate temperatures have been demonstrated to enhance the quality of Cu(In,Ga)(S,Se)_2_ films by reducing the density of deep defects, improving their microstructural and optoelectronic properties, resulting in improved performance of the resulting solar cells.^[^ ^21^, ^26^, ^42^, ^61^, ^62^, ^63^ ^]^ These reports indicate that the high Ga incorporation in Cu(In,Ga)S_2_ necessary to achieve a higher bandgap requires an elevated substrate temperature for the synthesis of high‐quality absorbers. In this investigation, variations of the first‐stage substrate temperature were employed to determine the optimal temperature and facilitate the enhanced intermixing of Ga and In to mitigate composition segregation in Cu(In,Ga)S_2_ films.

To demonstrate the influence of the first‐stage substrate temperature on mitigating the segregated Cu(In,Ga)S_2_ compositions, three absorbers designated T335, T385, and T435 were processed at first‐stage substrate temperatures of 335, 385, and 435 °C, respectively, while maintaining the second‐and‐third stage substrate temperatures at 590 °C (see Figure S1, Table S1, Supporting Information). The cross‐sectional scanning electron microscopy (SEM) micrographs of the three films presented in Figure S2 (Supporting Information) show that T335 and T385 feature large and compact grains extending to the back contact of the films, whereas T435 features smaller grain sizes and voids at the back contact. Absorbers processed under conditions similar to those used for T435 typically exhibited similar morphologies.

**Figure 1:**
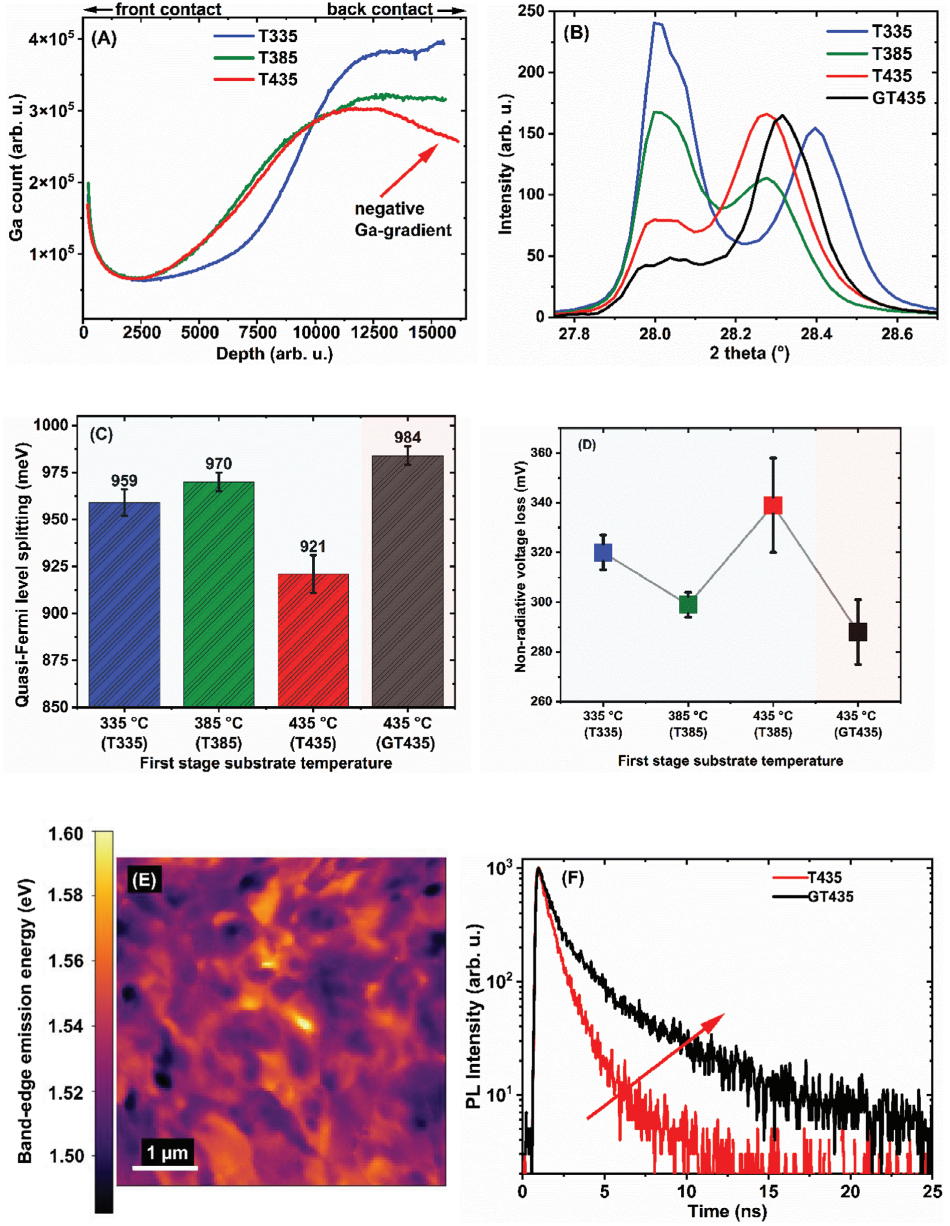
A) Secondary ion mass spectrometry profiles showing normalized Ga profiles for T335, T385, and T435. B) X‐ray diffractogram of the (112) chalcopyrite peak for absorbers with different first‐stage substrate temperatures. C) Quasi‐Fermi level splitting and D) non‐radiative voltage loss from photoluminescence quantum yield of T335, T385, T435 and GT435. E) Cathodoluminescence map of the near‐band‐edge emission energy of T435. F) Photoluminescence decay curves of T435 and GT435 at room temperature.

Secondary ion mass spectroscopy (SIMS) was conducted to examine the effect of varying first‐stage substrate temperatures on the Ga‐gradient. The SIMS depth profiles presented in **Figure** **1**A reveal that in T385 and T435, there is a smoother increase in Ga content between the front and back portions of the film, in comparison to T335. This suggests that increasing the first‐stage temperature resulted in a more gradual Ga gradient and reduced the compositional segregation in the films. On the other hand, the SIMS analysis of T435 in Figure 1A revealed a negative Ga gradient, indicating Ga‐depletion at the back contact. The consequences of this Ga‐depletion are analyzed in subsequent sections. Furthermore, the XRD analysis of the three Cu(In,Ga)S_2_ absorbers is presented in Figure 1B. The full XRD patterns and the positions of CuInS_2_ and CuGaS_2_ are shown in Figure S3 (Supporting Information). The XRD analysis revealed the reflection of two (112) peaks of the chalcopyrite crystal structure, corresponding to two distinct compositions. For T385 and T435, the peak at the higher angle, corresponding to the high Ga content, shifted to a lower angle. This shift indicates a reduced concentration of Ga at the back contact, which is consistent with the smoother Ga gradient and the findings from the SIMS analysis.

Photoluminescence (PL) spectroscopy was conducted on the Cu(In,Ga)S_2_ absorbers to analyze the optoelectronic properties of the films, the details of which are summarized in Table S2 (Supporting Information). A description of how these values were evaluated is presented in the Experimental Section. Figure 1C,D depict the QFLS and non‐radiative voltage loss with respect to the first‐stage substrate temperature. A minor increase in QFLS, due to a decrease in the non‐radiative voltage loss, was observed between T335 and T385. However, a significant decrease of ≈49 meV in the QFLS and an increase in the non‐radiative voltage loss by ≈30 meV occurred in T435. Time‐resolved photoluminescence (TRPL) measurements at room temperature (Figure S4, Supporting Information) showed that the minority carrier lifetime followed the trends of QFLS and non‐radiative voltage loss. Specifically, the lifetime decreased from (τ_1_: 835 ps and τ_2_: 5.01 ns) in T385 to (τ_1_: 617 ps and τ_2_: 1.86 ns) in T435, indicating a higher minority carrier recombination. Cathodoluminescence (CL) hyperspectral imaging was employed to map the lateral variation in the emission energy of T435 (Figure 1E). Across a 5 µm × 5 µm area, the near‐band‐edge (NBE) emission energy exhibited a mean value of 1.53 eV with a standard deviation of 10 meV. This represents a significant improvement compared to prior studies in which Cu(In,Ga)S_2_ absorbers were processed at lower first‐stage substrate temperature closer to T335, where the standard deviation of NBE reached 18 meV.^[^ ^64^ ^]^

Consequently, these results suggest that a more uniform Ga gradient from the higher first‐stage substrate temperature between T335 and T385 leads to improved optoelectronic quality. Although T435 also exhibited a smooth Ga gradient, the deterioration in the optoelectronic quality can be attributed to the Ga‐depletion toward the back contact, as observed in the SIMS profile in Figure 1A. This Ga‐depletion is detrimental to the device performance because of its two‐fold impact on charge carrier transport: 1) Enhanced back‐contact recombination: The gradient promotes a conduction band profile that favors electron diffusion toward the back contact. This phenomenon results in an increased back‐contact recombination. 2) Hindered electron collection: The negative gradient impedes the efficient diffusion of electrons toward the top of the film, where they are collected by external contact. This reduces the overall current‐collection efficiency of the device.^[^ ^29^, ^65^ ^]^

These findings seem to suggest that 385 °C is the optimal first‐stage temperature. Therefore, we proceeded to increase the Ga content in the first stage to achieve a higher bandgap. However, as shown in Figure S5 (Supporting Information), the effect of composition segregation became more pronounced in the films than in T385. Consequently, to take advantage of the similarly improved gradient and the homogeneity demonstrated in T435, a systematic approach was implemented to counteract the Ga depletion at the back contact by providing surplus Ga during the first stage of deposition for absorbers at 435 °C.

In the absorbers processed to counteract the Ga depletion, the sample with the lowest non‐radiative voltage loss, GT435, was processed at the first‐stage substrate temperature of 435 °C, but with a higher Ga flux than T435 during the first stage (Table S1, Supporting Information). The optimal Ga flux was 0.05 ± 0.01 nms^−1^, as the Cu(In,Ga)S_2_ films deposited with higher Ga flux exhibited lower optoelectronic quality, that is, an increase in the non‐radiative loss as presented in Table S3 (Supporting Information). The X‐ray diffractogram of GT435 together with T435 is presented in Figure 1B, where it is observed that there is a shift toward higher angles, indicating an overall higher Ga, and the intensity of the (112) peak corresponding to the (low Ga) front layer decreased in GT435 relative to T435. As reported by Klinkert et al. in Cu(In,Ga)Se_2_,^[^ ^66^ ^]^ such observation suggests a smoother gradient of the Cu(In,Ga)S_2_ composition in GT435. The SIMS profile for GT435 is not available in this report; however, as evidenced in the SIMS depth profiles of CR20 and CR07 (see Figure 3A below), both samples that will be discussed subsequently were processed using methods similar to GT435 (high first‐stage temperature and high Ga flux in the first stage, see Tables S1 and S2, Supporting Information), no indication of Ga‐depletion was observed. Compared to T435, GT435 showed a QFLS gain of ≈60 meV, a decreased non‐radiative voltage loss of ≈50 meV (Figure 1D), and a longer carrier lifetime (τ_1_: 783 ps and τ_2_: 4.40 ns), as shown in Figure 1F. These results indicate that the mitigation of the Ga depletion suppressed the back contact recombination, improved charge carrier extraction, and improved optoelectronic quality. The improved quality of the absorber with suppressed back contact recombination is similar to observations in Cu(In,Ga)Se_2_, where optoelectronic quality and carrier lifetime increased due to the passivation of back contact recombination.^[^ ^67^ ^]^

**Figure 2:**
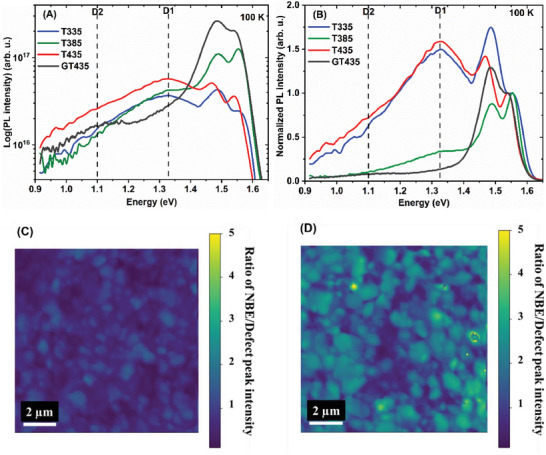
A) Low‐temperature photoluminescence spectra at 100 K for the Cu(In,Ga)S_2_ films (T335, T385, T435, and GT435) processed at different first‐stage substrate temperatures. B) Low‐temperature photoluminescence spectra normalized to the peak with the highest emission energy. C) Room temperature cathodoluminescence map of the ratio of the near‐band‐edge emission intensity to the defect emission peak at 1.3 eV of T435 and D) GT435.

Thus far, we have demonstrated that a higher first‐stage substrate temperature (with an appropriate Ga supply in the first stage) leads to an improved smoother Ga profile and reduced non‐radiative losses in the Cu(In,Ga)S_2_ absorbers under investigation. In a previous study^[^ ^19^ ^]^ we identified two deep defect photoluminescence emissions, that potentially increase the non‐radiative loss: D1 ≈1.3 eV and D2 ≈1.1 eV. The defects contributing to the D2 emission were found to dominate the stoichiometric and Cu‐rich films and to increase non‐radiative recombination. The films in the present study were all Cu‐poor, and as expected, the D1 emission dominated the D2 emission (see **Figure** **2**A,B). In particular, the two films with the lowest non‐radiative losses (T385 and GT435, see Figure 1D) showed a strongly reduced D1 emission. This observation indicates that, in the absence of the D2 defects, the D1 may provide the main path for non‐radiative recombination. There was no trend in the relative intensity of the D1 emission (Figure 2B) with the substrate temperature. The spectra also demonstrate that the Ga/In ratio during the first stage can influence the appearance of the D1 emission. We can only state that several process parameters influence the relative intensity of the D1 emission. A dedicated study that may also lead to a better identification of the defects involved is underway. We would like to investigate whether the strong difference in non‐radiative recombination between the two films with the highest first‐stage temperature (T435 and GT435), with and without increased Ga in the first stage, may be due to other inhomogeneities besides the reversed Ga gradient at the back contact. Therefore, cathodoluminescence mapping was performed on these two samples. Figure 2C,D shows the ratio of the near‐band edge intensity ≈1.55 eV to the intensity of the defect‐related emission ≈1.3 eV. A comparison of the maps of T435 and GT435 again shows that the near‐bandgap emission is higher than the defect emission, that is, the defect emission is lower with additional Ga during the first stage. It was also demonstrated that the lateral variation in this ratio was comparable for both samples. Therefore, we conclude that two factors contribute to the high non‐radiative loss in T435: the presence of deep defects that contribute to the D1 emission and the reversed Ga gradient at the back side, which increases the back‐side recombination.

These results demonstrate that a higher first‐stage substrate temperature is beneficial for mitigating composition segregation, which previously constrained the optoelectronic quality of wide‐bandgap Cu(In,Ga)S_2_ films. A mechanism underlying these improvements at higher temperatures requires a re‐examination of the probable origin of composition segregation from the Cu_2_S‐In_2_S_3_‐Ga_2_S_3_ pseudo‐ternary phase diagram.^[^ ^27^, ^36^ ^]^ The pseudo‐ternary phase reveals that Cu(In,Ga)S_2_ possesses two immiscible Cu‐poor phases, specifically the trigonal and cubic phases.^[^ ^27^, ^36^ ^]^ In the growth process of Cu(In,Ga)S_2_ films by the three‐stage deposition method, it is inevitable for the film to pass through this Cu‐poor phase during the second stage when Cu is introduced to form the chalcopyrite phase. It should be noted, however, that the pseudo‐ternary phase diagram was studied at room temperature.^[^ ^27^ ^]^ Therefore, we speculate that at higher temperatures, the formation or segregation of the trigonal and cubic phases is less pronounced, resulting in reduced composition segregation in the final Cu(In,Ga)S_2_ films.

### 2.3 Tuning Cu‐Excess for Higher Bandgap and Lower Bulk Recombination

During the three‐stage deposition process, the absorber undergoes a Cu‐rich phase in which Cu chalcogenide phases form. For the related selenides, it has been shown that the formation of the Cu_x_Se secondary phase plays a crucial role in the recrystallization process and facilitates grain growth.^[^ ^44^, ^49^, ^68^ ^]^ Additionally, studies have shown that Cu‐excess deposition significantly affects the bandgap minimum, absorber thickness, and optoelectronic performance.^[^ ^47^, ^48^ ^]^ Therefore, after establishing an optimal first‐stage substrate temperature for Cu(In,Ga)S_2_ absorbers exhibiting an optical bandgap of ≈1.56 eV at 435 °C (Table S2, Supporting Information), we investigated the effect of the Cu‐excess during the second stage on the optoelectronic quality of the absorbers.

The three‐stage process offers a particularly convenient and reliable way to control the Cu‐excess. At the end of the first stage, the film consists of (GaIn)S_x_. When Cu is deposited on this film, it starts forming the chalcopyrite structure first with a very high Cu deficiency.^[^ ^45^, ^49^ ^]^ During the second stage, an increasing amount of Cu is included in the film until it eventually reaches stoichiometric composition, subsequently becoming Cu‐rich. The Cu‐rich film becomes a 2‐phase structure with CuS_x_ forming on top of the film. As the emissivity of the Cu sulfide is higher than that of the chalcopyrite, this transition is observable in the output power of the heater and the effective temperature reading of the pyrometer,^[^ ^69^, ^70^, ^71^ ^]^ (see Figure 6 in the Experimental Section). Thus, we can determine the time from the beginning of the second stage to the (first) stoichiometric point (*T_bs_*). The time from this stoichiometric point to the end of the second stage (*T_as_*) can be controlled. At the end of the second stage, the Cu shutter is closed, no more Cu is provided to the sample, and In and Ga are provided again to make the final film Cu poor (after passing through the second stoichiometric point, which can again be detected from the power output and the pyrometer reading). Because the Cu flux during the second stage is constant, the ratio of the time after the stoichiometric point *T_as_* to the time before the stoichiometric point *T_bs_* is a measure of the excess Cu reached at the end of the 2^nd^ stage. In the following section, we investigate the influence of the excess Cu at the end of the 2^nd^ stage on the compositional, structural, and optoelectronic properties of the films. We would like to stress that the samples to be examined were deposited using the same process parameters (besides *T_as_*) as GT435, based on its superior homogeneity and optoelectronic quality. All the films discussed thus far had a Cu‐excess of 20%. Subsequently, the Cu‐excess varied between 6% (CR06) and 20% (CR20), as presented in Table S4 (Supporting Information); however, the final [Cu]/([In]+[Ga]) ratio of the films was maintained at ≈0.96. This is controlled by the duration of the third stage. Further details of all the absorbers, including the chemical composition acquired from energy‐dispersive X‐ray spectroscopy (EDX) and the optical bandgap, are presented in Tables S1 and S4 (Supporting Information). Expectedly, the variation of the Cu‐excess deposition time correlates with the thickness of the Cu(In,Ga)S_2_ absorbers,^[^ ^47^ ^]^ as reduced deposition time during the second and third stages results in a decrease in film thickness from 3.2 µm to 3.0 µm as shown in the SEM micrographs in Figure S6 (Supporting Information). The micrographs reveal distinct morphological characteristics for both films, with CR20 exhibiting larger top‐layer grains than CR07. This phenomenon is attributed to the higher Cu‐content in CR20, as Cu is known to contribute to the formation of larger grains and enhance the crystallinity of Cu(In,Ga)(S,Se)_2_ films.^[^ ^19^, ^44^, ^72^ ^]^

**Figure 3:**
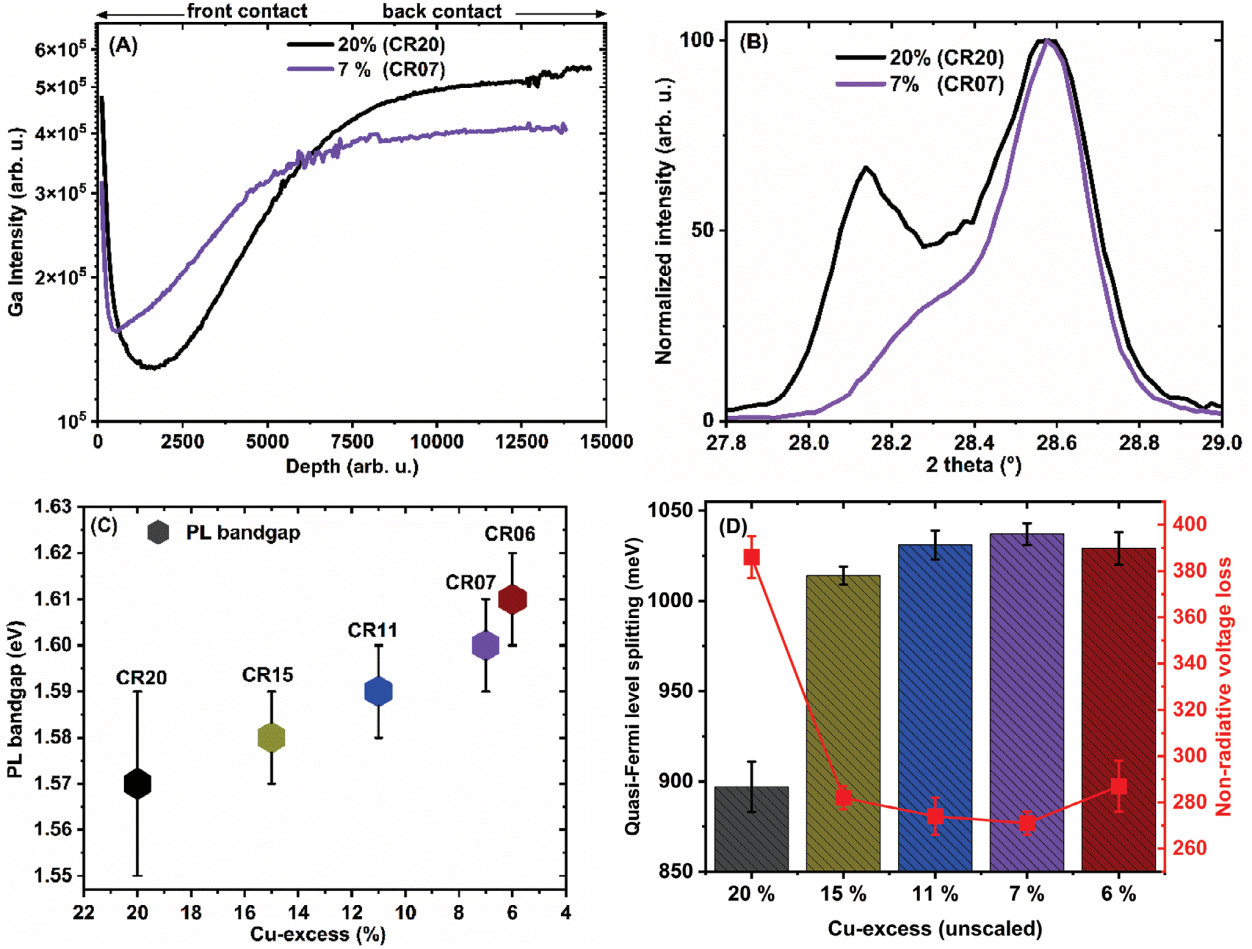
A) Ga depth profile acquired from secondary ion mass spectrometry measurements for CR20 and CR07 grown with different Cu‐excesses. B) Normalized X‐ray diffraction pattern around the (112) chalcopyrite peaks of CR20 and CR07. C) Optical bandgap of the absorbers determined from the photoluminescence maximum energy plotted against the percentage of Cu‐excess during growth of the absorbers. D) Quasi‐Fermi level splitting and non‐radiative voltage loss evaluated for absorbers grown with different Cu‐excess.

The SIMS depth profiles of Ga in the CR20 and CR07 absorbers are presented in **Figure** **3**A to observe the impact of varying Cu‐excess. As shown in Figure 3A, the notch width and position were particularly changed by the varied Cu‐excess. In CR07, with lower Cu‐excess, the notch is narrower, its Ga level is higher, and it is closer to the surface compared to CR20. A similar phenomenon, where varying Cu‐excess yields different bandgap grading profiles, has been reported in Cu(In,Ga)Se_2_.^[^ ^48^ ^]^ The shift of the notch closer toward the surface with decreasing Cu‐excess was attributed to the change in the duration of the third stage. Due to the higher diffusivity of In compared to Ga, In reaches and more readily intermixes with the Cu deposited during the second stage than Ga does.^[^ ^48^ ^]^ As a result, the relative proportion of In compared to Ga is higher within the bulk of the absorber. Furthermore, the SIMS profiles indicate a smoother Ga gradient with a lower Cu‐excess (CR07). This is also found in the X‐ray diffractograms in Figure 3B), where the second peak almost disappears, indicating a significantly reduced phase separation. Due to the high Ga level in the notch, lower Cu‐excess resulted also in the GGI ratio increasing from 0.20 (CR20) to 0.28 in (CR06) (see Table S4, Supporting Information) with a corresponding increase in the optical bandgap from 1.57 eV (CR20) to 1.61 eV (CR06) (Figure 3C).

The results presented in Figure 3D illustrate the QFLS and non‐radiative voltage loss determined from the absolute PL on the films, while Table S4 (Supporting Information) provides a comprehensive overview of the optoelectronic properties of the films. The results indicate that reducing the Cu‐excess from 20 to 7% resulted in a QFLS increase of ≈140 meV. However, at 6% Cu‐excess, a significant decrease in QFLS was observed, as illustrated in Figure 3D. Given that a higher bandgap contributes to the increase in the QFLS,^[^ ^73^, ^74^, ^75^, ^76^ ^]^ the non‐radiative voltage loss is additionally presented in Figure 3D to facilitate a more accurate comparison of the optoelectronic quality of the absorbers. Figure 3D shows that the non‐radiative voltage loss decreased from 386 mV in CR20 to 271 mV in CR07 and subsequently exhibited a slight increase to 287 mV in CR06. The decrease in non‐radiative loss is at least partly due to a longer minority carrier lifetime (see Figure S7, Supporting Information). The results of the optoelectronic evaluation indicate that under the conditions in which these absorbers were processed, a 7% Cu‐excess was optimal. In summary, the variation of Cu‐excess influenced the optical bandgap of the Cu(In,Ga)S_2_ films due to the alteration of the notch bandgap, and additionally affected the QFLS and the non‐radiative voltage loss. Interestingly, a lower Cu excess leads to better optoelectronic properties. The effect of varying the Cu excess on solar cell devices is not presented in this report, however, in Cu(In,Ga)Se_2_ films, it was reported that the variation of Cu‐excess did not have a significant impact on the performance of the solar cells fabricated on absorbers.^[^ ^47^, ^48^ ^]^ These results are noteworthy because they demonstrate the potential of increasing the bandgap of Cu(In,Ga)S_2_ absorbers without incorporating excess Ga during film deposition. Conventionally, in Cu(In,Ga)Se_2_, increasing Ga content deepens shallow defects, leading to enhanced non‐radiative recombination.^[^ ^77^ ^]^

### 2.4 Minimized Interface Losses in Cu(In,Ga)S_2_ with Tailored Zn_1‐x_Sn_x_O_y_ Buffer Layer

Now we use these optimized absorbers to prepare solar cells. In the first series, we optimize the buffer layer with a standard window layer. In the second step, we use then the buffer with the best solar cell efficiency and optimize the window layer.

We chose ZnSnO_x_ as the buffer layer, as we have previously shown that this buffer is preferable to other buffer materials.^[^ ^24^ ^]^ We have presented Cu(In,Ga)S_2_ solar cells with 14% PCE using a Zn_1‐ x_Sn_x_O_y_ (x = 0.18) buffer deposited via atomic layer deposition (ALD).^[^ ^24^ ^]^ The higher GGI ratio in the notch and at the surface in our optimized absorbers (CR07) requires an adjustment of the buffer composition to have proper band alignment at the absorber‐buffer interface to facilitate appropriate translation of QFLS into V_OC_.^[^ ^24^ ^]^ To determine the correct composition, we varied the Sn (x) concentration in the Zn_1‐x_Sn_x_O_y_ buffer between x = 0.14 and 0.30, which should result in different conduction band alignments at the interface.^[^ ^78^ ^]^ Before the Zn_1‐x_Sn_x_O_y_ deposition, the samples were immersed in either an aqueous ammonia solution (10 wt%) or deionized (DI) water for 30 s, as this has been shown to improve the FF of the final device (see Figure S8, Supporting Information). X‐ray photoelectron Spectroscopy (XPS) and Kelvin Probe Force Microscopy (KPFM) were performed on the sample surface to monitor the chemical changes that could arise from rinsing (Figure S9, Supporting Information). The KPFM maps of the as‐grown samples showed patches with lower work functions, whereas XPS indicated the presence of Na from the soda‐lime glass at the Cu(In,Ga)S_2_ surface. Karami et al. previously reported that Na clusters can form at interfaces and grain boundaries of Cu(In,Ga)Se_2_.^[^ ^79^ ^]^ Therefore, it is probable that those regions with lower work function are similar to the clusters observed by Karami et al, which are rich in Na and consequently altered the electronic property of the surface.^[^ ^79^ ^]^ These areas of low work function in the KPFM maps were eliminated after rinsing the sample with DI water and Ammonia, and XPS did not detect Na at the surface. The results from XPS and KPFM suggest that a Na‐compound could be removed effectively with the DI water and ammonia treatment, potentially explaining the FF losses in the devices when no cleaning was performed. A bi‐layer window, consisting of a high resistance i‐layer and a transparent conductive oxide (TCO), i.e., undoped ZnO and Al:ZnO layer was deposited on the top of Zn_1‐x_Sn_x_O_y_ buffer layer to complete the solar cells in this first series for buffer optimization. The detailed procedure for the buffer and window deposition is described in the Experimental Section.

**Figure 4:**
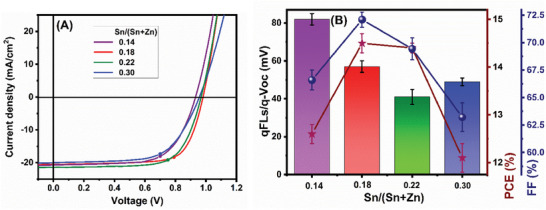
A) Measured current‐voltage characteristics of Cu(In,Ga)S_2_ devices with different Sn contents in the Zn_1‐x_Sn_x_O buffer layers. B) Interface V_OC_ deficit bar chart of Cu(InGa)S_2_ devices, scatter plot of power conversion efficiency (red, right axis), fill factor (blue, second right axis), versus different Sn contents in the Zn_1‐x_Sn_x_O buffer layers.

The current–voltage (J–V) characteristics of the Cu(In,Ga)S_2_ devices with different Sn contents in the Zn_1‐x_Sn_x_O buffer layers are shown in **Figure** **4**A. Absorber films processed in similar conditions to GT435 described in the preceding section were used as absorbers in this investigation. Therefore, we can assume the same optoelectronic quality of the absorber as measured by QFLS. Figure 4B illustrates the difference between QFLS/q obtained from PL quantum yield (PLQY) measurements and V_OC_ obtained from J–V measurements as a function of Sn content x, as measured by EDX. The interface V_OC_ deficit, defined as the difference between the QFLS/q and V_OC_,^[^ ^80^ ^]^ initially decreases with increasing Sn concentration in the Zn_1‐x_Sn_x_O_y_ buffer until x  =  0.22, reaching ≈40 meV and then increases slightly at higher Sn concentrations. The lowest interface V_OC_ deficit is comparable to the optimal values obtained for absorbers with lower surface Ga, as used in our earlier study.^[^ ^24^ ^]^ However, it is noteworthy that this interface V_OC_ deficit value in the higher bandgap absorbers is achieved with a slightly higher Sn concentration (x = 0.22) compared to x = 0.18 used in our previous study for the Zn_1‐x_Sn_x_O_y_ buffer.^[^ ^24^ ^]^ The superior interface quality with Zn_1‐x_Sn_x_O_y_ x = 0.22 buffer is potentially attributed to improved conduction band alignment at the Cu(InGa)S_2_/ Zn_1‐x_Sn_x_O_y_ interface, as the conduction band minimum of Zn_1‐x_Sn_x_O_y_ x = 0.22 is reported to be higher (i.e., lower electron affinity) than the conduction band minimum of Zn_1‐x_Sn_x_O_y_ x = 0.18 buffer.^[^ ^78^ ^]^ Figure 4B also shows the PCE of the cells. The cell with the lowest interface loss (x = 0.22) does not exhibit the highest efficiency. This can be understood from the decrease in the FF, which is indicated by the blue dots in Figure 4B. Here we have used a standard ZnO film as the i‐layer in the cells. This is not the ideal partner for a high‐conduction band buffer, as discussed in detail by Sood et al.^[^ ^25^ ^]^ The loss in FF can be mitigated by also using an i‐layer with a higher conduction band minimum, for example, ZnMgO^[^ ^25^ ^]^ or Al:ZnMgO.

### 2.5 Mg‐Doped Window Layer Optimized for High‐Efficiency Cu(In,Ga)S_2_ Solar Cells

Therefore, to enhance the FF and the PCE, we substitute the ZnO i‐window layer with an Al:Zn_1‐ y_Mg_y_O window layer. Considering that Zn_1‐x_Sn_x_O exhibits a higher conduction band minimum than ZnO,^[^ ^25^ ^]^ the i‐ZnO layer could potentially introduce an electrical barrier for the injected electrons, potentially resulting in a decrease in the FF. In previous research, this phenomenon was observed in both simulations and experiments when combining a ZnMgO buffer with a ZnO i‐layer.^[^ ^25^ ^]^

To optimize the conduction band alignment at the interface, the ratio of Mg “y” within the Al:Zn_1‐ y_Mg_y_O layer was adjusted, ranging from 0.19 to 0.30, to minimize FF loss while maintaining the Zn_1‐x_Sn_x_O buffer composition constant at the optimal x = 0.22. The fabrication process of the devices on the absorbers involved the deposition of a 70 nm thick Al:Zn_1‐y_Mg_y_O window layer, followed by a TCO Al:ZnO layer of ≈350 nm to construct the solar cells. Additionally, a reference solar cell was fabricated with 70 nm ZnO and 350 nm Al:ZnO window layers, utilizing the same buffer composition.

**Figure 5:**
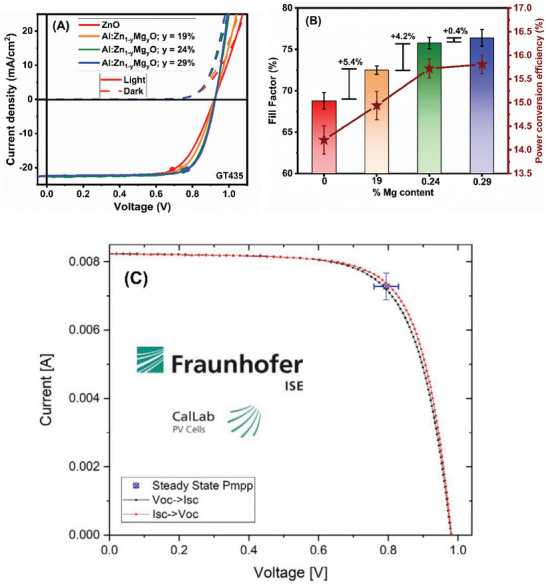
A) Measured current‐voltage characteristics of Cu(InGa)S_2_ devices with different Mg contents in the Al:Zn_1‐ x_Mg_x_O i‐window layer. B) Bar chart of the fill factor (scale on the left) and power conversion efficiency scatter chart (scale on the right) of the devices as a function of the Mg content in the Al:Zn_1‐y_Mg_y_O window layer. C) Certified current–voltage current–voltage curve of the best Cu(InGa)S_2_ device with 0.22 Sn ratio in the buffer and 0.29 Mg ratio in the i‐layer and with anti‐reflection coating.

As anticipated from previous simulations,^[^ ^25^ ^]^ the substitution of ZnO with Al:ZnMgO results in a substantial improvement in FF and, therefore, in PCE (**Figure** **5**A,B). The FF showed an absolute increase of 5.4% compared to that of pure ZnO for the device with 19% Mg, and a relative increase of 10.2% compared to ZnO with 29% Mg content in the window layer. Moreover, the increase in FF saturates above ≈24% Mg content, as a marginal improvement was observed from 24 to 29% Mg content. Mg contents exceeding 29% in the window layer were also investigated, but they led to significant deterioration in the FF. These findings suggest that Al:ZnMgO with a Mg content of 29% is the optimum composition for the window layer.

Finally, our champion Cu(In,Ga)S_2_ absorber film was grown using a deposition process similar to that used for CR07. The optoelectronic characteristics of the champion film are presented in Table S5 (Supporting Information). Utilizing the Zn_1‐x_Sn_x_O_y_ buffer layer, optimized window layer, and MgF_2_ anti‐reflection coating (ARC), we obtained a device with an in‐house PCE of 16.1%, with an independently certified PCE of 14.81%, and a V_OC_ of 981 mV on a solar cell with an active area of 0.41 cm^2^. The J‐V curve obtained from the independent certification of the champion device is shown in Figure 5C and in Figure S10A (Supporting Information) along with the in‐house measurement, while the solar cell parameters are presented in **Table** **1**, and the integrated short‐circuit current density (J_SC_) and external quantum efficiency (EQE) curves are shown in Figure S10B (Supporting Information). The relatively large difference between in‐house and certified efficiency is because we report active area efficiency for the in‐house measurement and that this cell has a non‐optimized Ni‐Al grid on top that results in ≈10% of the shaded area. The solar cell bandgap of this device is 1.63 eV, as determined by the EQE inflection point. This value represents the highest PCE for any Cu(In,Ga)S_2_ device with a bandgap exceeding 1.6 eV (as shown in Figure S11, Supporting Information), and the V_OC_ is the highest certified value for any Cu(In,Ga)S_2_ device. This achievement is particularly noteworthy because it demonstrates the feasibility of achieving high efficiency in wide‐bandgap single‐junction Cu(In,Ga)S_2_ solar cells. This advancement paves the way for the potential application of this technology as a top cell for tandem solar cell configurations.

**Table 1:** The solar cell parameters for the champion Cu(In,Ga)S_2_ solar cell were obtained from in‐house measurements and independent certification by the Fraunhofer ISE with an active area of 0.41 cm^2^.

Device measurement	V_OC_ [mV]	I_SC_ [mA]	FF [%]	PCE [%]
In‐house	966	8.23	75.76	16.1 ± 0.1
Fraunhofer ISE	981	9.31	72.6	14.81 ± 0.90

A comparative analysis of the J_SC_, and the product of V_OC_ and FF compared to the respective Shockley‐Queisser J_SC_, V_OC,_ and FF values denoted here as $J_{\mathrm{SC}}^{\mathrm{SQ}}$, $V_{\mathrm{OC}}^{\mathrm{SQ}}$, and ${\mathrm{FF}}_{\mathrm{OC}}^{\mathrm{SQ}}$ for Cu(In,Ga)S_2_ devices with PCE above 15% are shown in Figure S12 (Supporting Information). The chart also incorporates data from this study and the state‐of‐the‐art (Ag,Cu)(In,Ga)Se_2_^[^ ^17^ ^]^ device (for comparison). As evident in Figure S12 (Supporting Information), the Cu(In,Ga)S_2_ devices under investigation primarily suffer from lower V_OC_ and FF values than the record selenide device. Even the highest Cu(In,Ga)S_2_ devices with 16% PCE^[^ ^22^ ^]^ exhibited ≈25% lower V_OC_ x FF/$V_{\mathrm{OC}}^{\mathrm{SQ}}$ x ${\mathrm{FF}}_{\mathrm{OC}}^{\mathrm{SQ}}$ when compared to Cu(In,Ga)Se_2_ devices, indicating a significant potential for improvement in the device PCE for Cu(In,G)S_2_. Furthermore, the optimization of the window layers and ARC is expected to improve the J_SC_/$J_{\mathrm{SC}}^{\mathrm{SQ}}$ ratio.

## 3 Conclusion

This study demonstrates a significant advancement in achieving high‐efficiency wide‐bandgap Cu(In,Ga)S_2_ solar cells by overcoming the inherent challenge of composition segregation through the optimization of a three‐stage co‐evaporation process by employing a higher substrate temperature during the first stage. A higher first‐stage substrate temperature promoted better intermixing of Ga and In, leading to a marked reduction in composition segregation and a smoother bandgap profile within the absorber layer. The sample also showed better lateral homogeneity, as confirmed by CL imaging, which revealed more uniform near‐band‐edge emission energy than that of samples from earlier studies. Nevertheless, a trade‐off was identified wherein a higher first‐stage substrate temperature counteracted the benefit of an improved bandgap profile by depleting Ga near the back contact, thereby increasing non‐radiative recombination at the metal contact. This problem was effectively addressed by systematically providing surplus Ga during the first stage, thereby ensuring sufficient Ga near the back contact at high substrate temperatures. The combined effect of minimized composition segregation and mitigated depletion of Ga at the back contact resulted in several improvements, namely, reduced non‐radiative voltage loss, which contributed to QFLS values ≈1 eV, along with a longer carrier lifetime.

Furthermore, this study demonstrated the influence of Cu‐excess during the second stage on the bandgap within the notch (a critical region for light absorption). These findings indicate that by decreasing the Cu‐excess, it was possible to regulate the excess Ga required to achieve a higher bandgap. Additionally, a lower Cu‐excess resulted in reduced non‐radiative voltage loss and QFLS values exceeding 1 eV.

Finally, to maximize the conversion of the QFLS into V_OC_ in these higher‐bandgap devices, we optimized the buffer and bilayer window layers. This involved employing an atomic‐layer‐deposited Zn_1‐x_Sn_x_O_y_ buffer layer with a sputtered Al:ZnMgO i‐layer and Al:ZnO TCO. This optimization resulted in a substantial reduction in the interface V_OC_ deficit and an improvement in the FF. Consequently, we achieved a Cu(In,Ga)S_2_ device with a notable in‐house PCE of 16.1% and a certified PCE of 14.8%, with a certified record V_OC_ of 981 mV in a device with a bandgap of 1.63 eV. These findings demonstrate the potential for the application of pure sulfide Cu(In,Ga)S_2_ solar cells in high‐efficiency tandem configurations.

## 4 Experimental Section

### Cu(In,Ga)S_2_ Absorber Deposition Process

**Figure 6:**
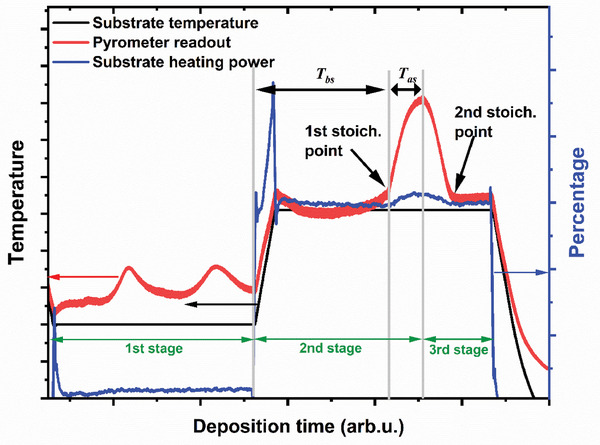
Typical three‐stage deposition profile of a Cu(In,Ga)S_2_ absorber showing the evolution of the substrate temperature, pyrometer readout, and substrate heating power at different steps during absorber growth. T_bs_ is the time between the start of Cu deposition and the first stoichiometric point during the second stage while T_as_ is the time in which Cu‐excess is deposited after the first stoichiometric point.

The Cu(In,Ga)S_2_ absorbers were fabricated using a physical vapor deposition (PVD) system from Plassys Bestek. Notably, the sulfur source for the deposition process was provided by Nano4energy and Gencoa. The three‐stage co‐evaporation process, illustrated in Figures 6 and S1 (Supporting Information), was used to fabricate the Cu(In,Ga)S_2_ absorbers on a Mo‐sputtered soda lime glass substrate. In the first stage, the substrate temperature was set between 335–435 °C, and then the samples were heated for 30 min under a sulfur atmosphere for a presulfurization step. The source temperatures of In and Ga with the corresponding elemental fluxes are presented in Table S1 (Supporting Information). Subsequently, In and Ga source shutters were opened under the sulfur environment to deposit a (In_x_Ga_1‐x_)_2_S_3_ layer of ≈1.7 µm thickness. In the second stage of the deposition process, the In and Ga shutters were closed, the Cu shutter was opened, and the substrate temperature increased to 590 °C at a rate of 20 °C min^−1^. The deposition continued until the [Cu]/[III] ratio exceeded the stoichiometric composition, forming Cu(In,Ga)S_2_ and secondary Cu_2_S phases. This was marked by an increase in the apparent surface temperature measured by the pyrometer in the system, as illustrated in **Figure** **6**.

In the third stage, Cu deposition was halted, and In and Ga were deposited again by opening the source shutters. The duration of the third stage of deposition determines the final [Cu]/[III] composition of the resulting Cu(In,Ga)S_2_ film. After achieving the desired composition, the In and Ga shutters were closed, and the sample was cooled to 200 °C at a rate of 20 °C min^−1^ under a sulfur atmosphere. The total deposition time varies between 140–157 min, 45–60 min for the first stage, 75 min for the second stage, and 20–22 min for the third stage. The real temperature measured on the substrate was calibrated against the known softening temperature of the soda‐lime glass (SLG) substrate used in this work, and the set heater temperature of the substrate heater at which the softening occurs in the deposition system.

### Buffer Layer Deposition

The Zn_1‐x_Sn_x_O_y_ buffer layer was deposited at 120 °C by atomic layer deposition in a Microchemistry F‐120 ALD reactor. Diethyl zinc (DEZ), tetrakis (dimethylamino) tin (TDMASn), and DI water (as co‐reactants) were used as precursors, with N_2_ as the carrier gas. Films with different [Sn]/[Zn+Sn] ratios were obtained using a super‐cycle approach with various ZnO:SnO_x_ pulse ratios. To obtain [Sn]/[Zn+Sn] compositions of 0.14, 0.18, 0.22, and 0.30, ZnO:SnO_x_ pulse ratios of 2:1, 3:2, 1:1, and 2:3 were used, respectively.

The [Sn]/[Zn+Sn] compositions of the films were measured using X‐ray fluorescence on soda‐lime glass pieces from the batch. Barring a few Cu(In,Ga)S_2_ absorbers, before buffer deposition, the absorbers were dipped in either an aqueous ammonia solution (10 wt %) or deionized water for 30 s, followed by blow‐drying using N_2_ gas and immediately transferred into the atomic layer deposition reactor.

### Window Layer Deposition

Magnetron sputtering was used to deposit ZnO, Al:ZnMgO, and Al:ZnO window layer using 2inch undoped ZnO, 2inch 2 wt% Al_2_O_3_ doped MgO target, and 2inch 2 wt% Al_2_O_3_ doped ZnO target. ZnO target was sputtered at 125 W for 33 min to deposit ZnO window to get the desired thickness of 70 nm. For the Al:ZnMgO window deposition the Al:MgO target was co‐sputtered at set power between 6080 W together with ZnO target at 125 W for ≈30 min to get a desired thickness of ≈70 nm. All the processes were performed at a partial pressure of 1.0 mTorr argon (99.99%) and maintained using a mass flow controller.

### Energy Dispersive X‐Ray Spectroscopy

Energy dispersive X‐ray spectroscopy was conducted in Zeiss EVO10. The bulk and near‐surface stoichiometries of the as‐grown absorbers were determined using EDX spectroscopy at operating voltages of 20 and 7 kV, respectively. For the buffer window layer stoichiometry, EDX spectroscopy was performed at an operating voltage of 7 kV.

### Photoluminescence Measurements

The optoelectronic properties of the absorbers were studied in a custom‐built setup using absolute intensity‐calibrated room‐temperature photoluminescence measurements of bare absorbers. The excitation source was a 405 nm wavelength continuous‐wave semiconductor laser. The luminescence from the absorbers was collected by mirrors, focused on a collection fiber, spectrally resolved by a monochromator, and detected by a Si‐CCD array. For intensity calibration, spectral and intensity corrections were applied to the raw data, using a calibrated lamp and a power meter. The QFLS was determined by evaluating the PLQY, determined from the integrated emission peak at the band‐edge at an excitation photon flux density equivalent to 1 sun.^[^ ^75^, ^76^ ^]^ The bandgap of the absorbers was determined from the maximum of the PL emission peak. From the PLQY, the deficit from the ideal Shockley–Queisser V_OC_ is the QFLS,^[^ ^73^, ^76^, ^81^ ^]^ as shown below:

(1) $$QFLS=qV_{OC}^{SQ}+k_{B}T\ln\left(\mathrm{PLQY}\right)$$

The non‐radiative voltage loss (*nrad_loss_*)  was defined using the difference between the ideal Shockley–Queisser V_OC_ and the QFLS:

(2) $$nrad_{loss}\;\approx k_{B}T\ln\left(\mathrm{PLQY}\right)$$

Time‐resolved photoluminescence (TRPL) measurements were conducted using a commercial time‐correlated single photon counting (TCSPC) system from Edinburgh Instruments, LifeSpec II Lifetime Spectrometer). The excitation source was a pulsed laser operating at a wavelength of 638 nm and a repetition rate of 20 MHz, ensuring an excess carrier concentration below the equilibrium hole density. PL emission was detected by a high‐speed photomultiplier with an integrated amplifier.

### Cathodoluminescence Measurements

Cathodoluminescence hyperspectral mapping was performed in an Attolight Allalin 4027 Chronos dedicated CL‐SEM with 150 l/mm and 500 nm blazed grating. All measurements were taken at room temperature with an electron beam acceleration voltage of 10.0 kV and a current of 10 nA with 90% of the beam energy being deposited within a depth between ≈400 nm according to Monte Carlo CASINO simulations.^[^ ^82^ ^]^ All data treatment was performed using the Python libraries hyperspy^[^ ^83^ ^]^ and lumispy.^[^ ^84^ ^]^

### Device Characterization

A commercial solar simulator from OAI was used for device characterization. The J–V curves of the devices were measured under air mass 1.5 global illumination intensity 1000 W m^−2^ at a temperature of 25 °C using a Xenon short‐arc lamp AAA‐Standard solar simulator together with a four‐terminal J–V source measurement unit. A certified Si reference cell was used to calibrate the lamp intensity at 1000 W m^−2^. The devices were measured in the forward sweep direction from −0.5 to 1.2 V with a sweep speed of 50 mVs^−1^. The EQE of the devices was measured using illumination from halogen and xenon lamps, grating monochromator, and chopper. Certified Si and InGaAs reference diodes were used for calibration.

### Kelvin Probe Force Microscopy (KPFM) and X‐Ray Photoelectron (XPS) Measurements

KPFM and XPS were used to investigate the effect of rising the sample surface with DI water and ammonia. To preserve the chemical properties on the surface, the rising was done in the anti‐chamber of an N_2_ glovebox. The samples were then transferred to the XPS and KPFM using an N_2_ transfer system to avoid any air exposure. The KPFM measurements were done under an N2 environment using a Nanoscope V (Veeco instruments) operated in Frequency Modulation (FM‐ KPFM) mode. Topography and the work function maps were acquired simultaneously in a single pass with the help of an external lock‐in amplifier (Zurich instruments). An AC voltage of 1 V and at a frequency of 856 Hz was used to modulate the KPFM signal. The used probe was the 240AC‐PP (OPUS by MikroMasch) with a resonance frequency between 70 and 90 kHz. XPS measurements were carried out using the EA‐15 energy analyzer (Prevac). The Al‐Kα (1486.6 eV) excitation energy was used at a power of 375 W and kept a main chamber pressure at 1.7 × 10^−10^ mbar. The samples were qualitatively analyzed by using the high‐resolution spectra from elements Cu, In, Ga, S, Na, and O.

### Statistical Analysis

The detailed results and values of this study, presented in the Supporting Information, are presented as $\bar{x}\pm \mathrm{SD}$, where $\bar{x}$ and SD and are the mean and standard deviation, respectively. The SD was evaluated by Equation (3), where n and *x_i_* are the sample size and values of the data set respectively.

(3) $$SD=\sqrt{\frac{1}{n-1}\sum_{i=1}^{n}\left(x_{i}-{\bar{x}}^{2}\right)}$$

## Conflict of Interest

The authors declare no conflict of interest.

## Author Contributions

D.A. and M.S contributed equally to this work. D.A., M.S., and S.S. conceived ideas, designed experiments, and wrote the first draft of the manuscript. S.S. was responsible for funding acquisition and project supervision. D.A. prepared samples and performed PL measurements, optoelectronic analyses, XRD, and EDX characterizations. M.S. performed buffer layer deposition, electrical measurements, and device fabrication. A.V.O. contributed to sample preparation, optoelectronic analyses, and electrical measurements. T.T. and A.H. contributed to buffer layer deposition. A.V. performed TRPL measurements. E.V.L. and A.R. performed and analyzed XPS & KPFM measurements. Y.H., G.K., and R.O. performed and analyzed CL measurements. M.M. contributed to buffer layer deposition & device fabrication. All authors participated in the discussion of data and results, and reviewed and edited the draft of the manuscript.

## Supporting information



Supporting Information

## Data Availability

The data that support the findings of this study are available in the supplementary material of this article.
