# Supplementary material for: Wide‐Bandgap Cu(In, Ga)S2 Solar Cell: Mitigation of Composition Segregation in High Ga Films for Better Efficiency
Source: Small. 2025 Jan 8;21(8):2405221. doi: 10.1002/smll.202405221 (PMC11855247; doi:10.1002/smll.202405221)
Supplement: Supplementary file 1 — Supporting Information [file SMLL-21-2405221-s001.pdf]

NANO · MICRO
small

Supporting Information

for *Small*, DOI 10.1002/smll.202405221

Wide-Bandgap Cu(In, Ga)S_2 Solar Cell: Mitigation of Composition Segregation in High Ga Films for Better Efficiency

Damilola Adeleye, Mohit Sood, Arivazhagan Valluvar Oli, Tobias Törndahl, Adam Hultqvist, Aline Vanderhaegen, Evandro Martin Lanzoni, Yucheng Hu, Gunnar Kusch, Michele Melchiorre, Alex Redinger, Rachel A. Oliver and Susanne Siebentritt**

Wide Bandgap $Cu(In,Ga)S_2$ Solar Cell: Mitigation of Composition Segregation in High Ga Thin Films for Better Efficiency

Damilola Adeleye[1,], Mohit Sood[1], Arivazhagan Valluvar Oli, Tobias Törndahl, Adam Hultqvist, Aline Vanderhaegen, Evandro Martin Lanzoni, Yucheng Hu, Gunnar Kusch, Michele Melchiorre, Alex Redinger, Rachel A. Oliver, Susanne Siebentritt[*]*

D. Adeleye, M. Sood, A. Valluvar Oli, A. Vanderhaegen, E. M. Lanzoni, M. Melchiorre, A. Redinger, S. Siebentritt

Department of Physics and Materials Science, University of Luxembourg, L-4365 Esch-sur-Alzette, Luxembourg

Corresponding authors: damilola.adeleye@helmholtz-berlin.de, susanne.siebentritt@uni.lu

T. Törndahl, A. Hultqvist

Department of Materials Science and Engineering, Uppsala University, Uppsala 75103, Sweden

Y. Hu, G. Kusch, R. Oliver

Department of Materials Science and Metallurgy, University of Cambridge, Cambridge, UK

[1] These authors contributed equally

Supporting Information

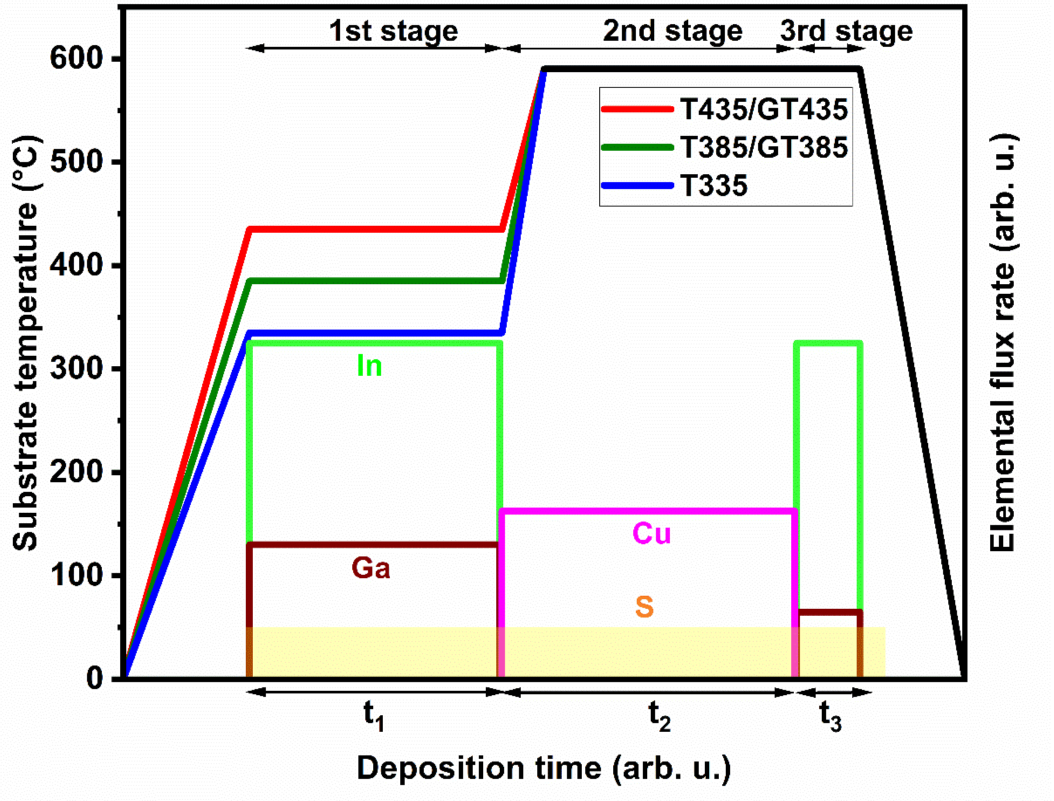

Figure S1. *The three-stage deposition profile used in processing the Cu(In,Ga)S_2 films at different first-stage substrate temperature.* t_1, t_2 *and* t_3 *represent the first, second and third stage deposition times.*

Table S1. Cu, In and Ga fluxes used during the deposition of the Cu(In,Ga)S_2 absorbers

**Sample**	***1st and 3rd stage In flux* (nm/s)**	***1st stage Ga flux* (nm/s)**	***2nd stage Cu flux* (nm/s)**	***3rd stage Ga flux* (nm/s)**
T335	0.20±0.02	0.04±0.01	0.05±0.001	0.02±0.005
T385	0.20±0.02	0.04±0.01	0.05±0.001	0.02±0.005
T435	0.20±0.02	0.04±0.01	0.05±0.001	0.02±0.005
GT435	0.20±0.02	0.05±0.01	0.05±0.001	0.02±0.005
CR20	0.20±0.02	0.06±0.01	0.05±0.001	0.02±0.005
CR15	0.20±0.02	0.06±0.01	0.05±0.001	0.02±0.005
CR11	0.20±0.02	0.06±0.01	0.05±0.001	0.02±0.005
CR07	0.20±0.02	0.06±0.01	0.05±0.001	0.02±0.005
Certified device	0.20±0.02	0.05±0.01	0.05±0.001	0.02±0.005

Table S2. The chemical compositions evaluated from energy-dispersive X-ray spectroscopy analyses, and the optical properties of the $Cu(In,Ga)S_2$ absorbers deposited at different first stage substrate temperatures.

Sample name	First stage substrate temperature (°C)	CGI ratio @ 20 kV	GGI ratio @ 20 kV	GGI ratio @ 7kV	E_g^{PL} (eV)	QFLS (meV)	$nrad_{loss}$ (meV)
T335	335	0.95±0.01	0.11±0.01	0.19±0.01	1.55±0.01	959±7	320±7
T385	385	0.97±0.02	0.12±0.01	0.19±0.01	1.55±0.01	970±5	299±5
T435	435	0.95±0.01	0.11±0.01	0.20±0.02	1.55±0.02	921±10	339±19
GT435	435	0.96±0.02	0.14±0.01	0.16±0.01	1.56±0.01	984±5	288±13

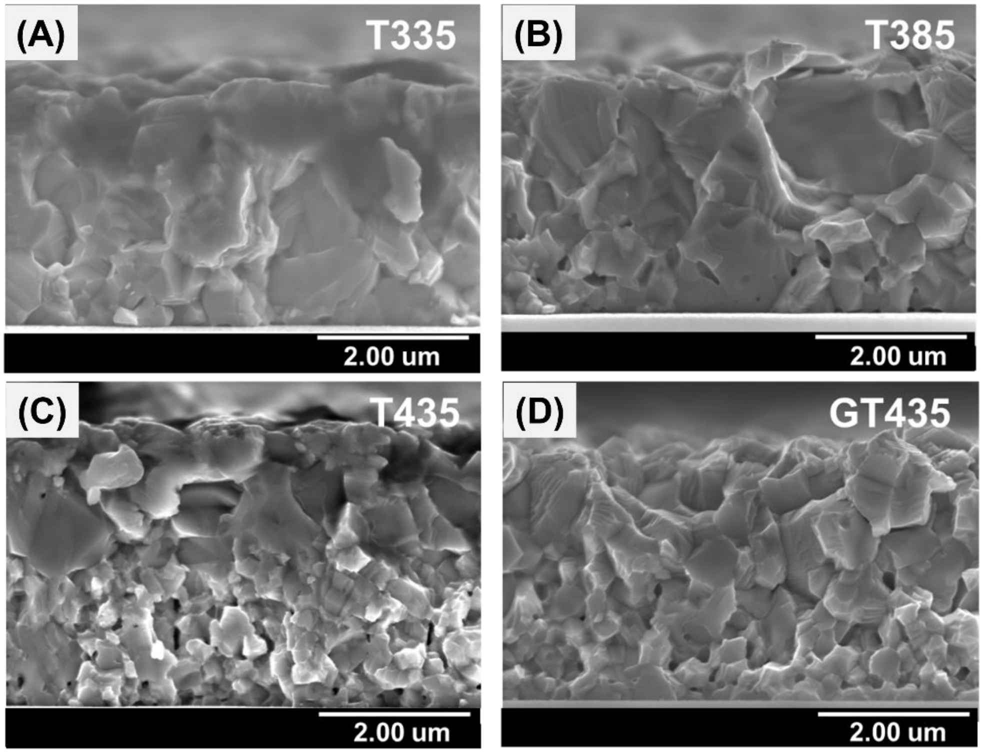

Figure S2. *Cross-sectional scanning electron microscopy of the films grown at first-stage temperature of A) 335 °C, B) 385 °C, C) 435 °C, and D) 435 °C with surplus first stage Ga-flux.*

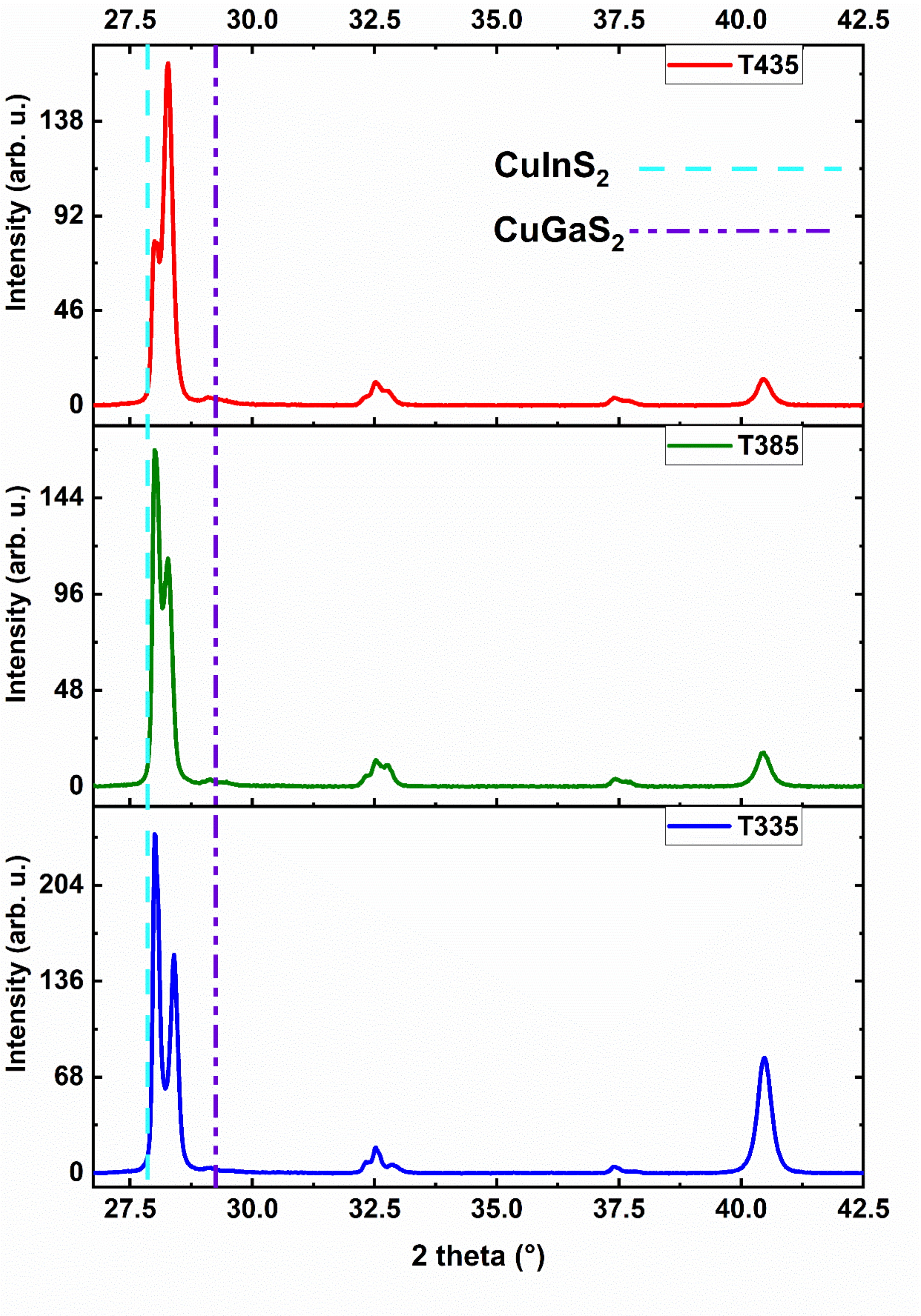

Figure S3. *X-ray diffraction diffractogram showing the peak position of the dominant chalcopyrite phase of the Cu(In,Ga)S_2 films T335, T385 and T435. The peak positions for $CuInS_2$ and $CuGaS_2$ are also highlighted by the dashed lines.*

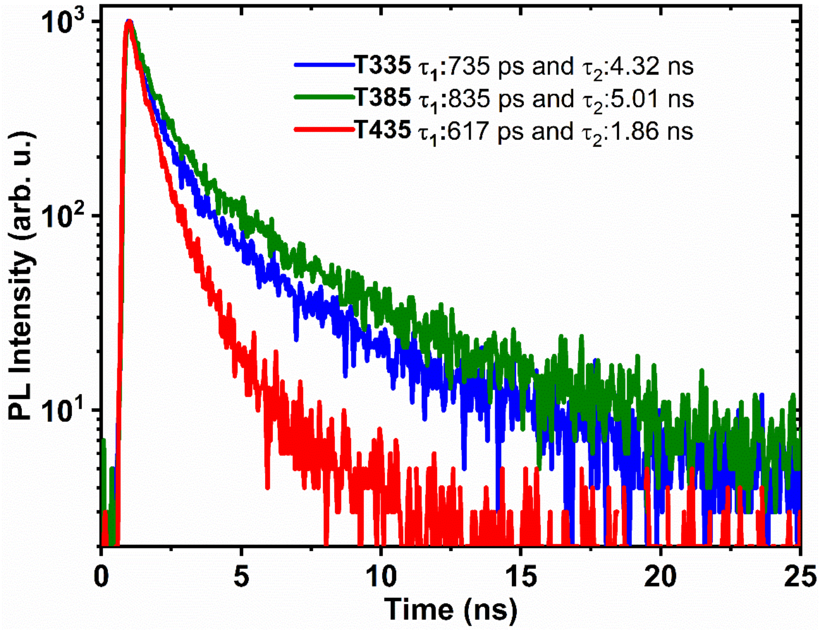

Figure S4. *Transient photoluminescence decay measurement for T335, T385, and T435.*

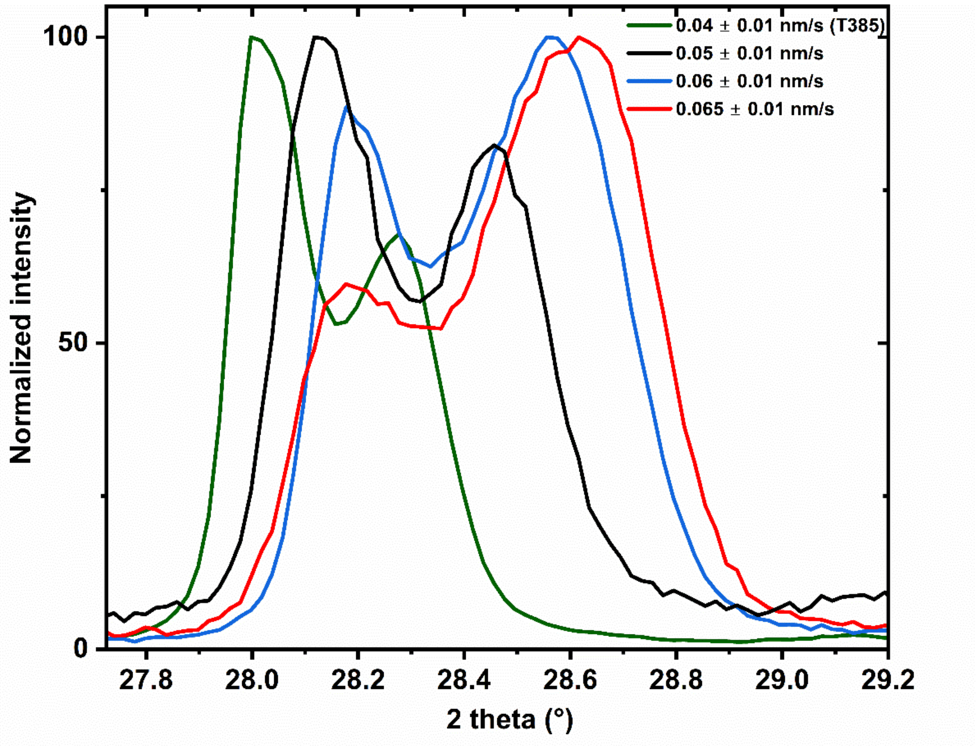

Figure S5. *X-ray diffraction diffractogram of the (112) chalcopyrite peak for absorbers, including T385, processed at the first stage substrate temperature of 385°C but with different first-stage Ga flux of 0.04±0.01, 0.05±0.01, 0.06±0.01 and 0.065±0.01nms*$^{-1}$.

Table S3. Optoelectronic quality for different absorbers grown at deposited at first-stage substrate temperature of 435°C with various first stage Ga fluxes.

Sample	1st stage Ga flux	E_g^{PL} (eV)	QFLS (meV)	$nrad_{loss}$ (meV)
T435	0.04±0.01	1.55±0.02	921±10	339±19
GT435	0.05±0.01	1.56±0.01	984±5	288±13
Test1	0.06±0.01	1.57±0.01	911±14	386±9
Test2	0.065±0.002	1.59±0.01	1021±14	317±12

Table S4. Cu-excess deposition time and chemical composition extracted from energy-dispersive X-ray spectroscopy analysis for the $Cu(In,Ga)S_2$ absorbers deposited with varied Cu-excess. All the absorbers in this series were processed with first stage substrate temperature of 435°C.

Sample	T_{as} (mins)	$\left(\frac{T_{as}}{T_{bs}}\right)$ (%)	Average CGI ratio	Average GGI ratio	E_g^{PL} (eV)	QFLS (meV)	$nrad_{loss}$ (meV)
CR20	13:00	20	0.97±0.02	0.20±0.02	1.57±0.01	911±14	386±9
CR15	09:30	15	0.96±0.02	0.23±0.01	1.58±0.01	1014±5	282±5
CR11	06:00	11	0.96±0.01	0.24±0.01	1.59±0.01	1031±8	274±8
CR07	04:30	7	0.96±0.01	0.25±0.01	1.60±0.01	1037±6	271±5
CR06	04 :00	6	0.97±0.02	0.26±0.02	1.61±0.01	1028±9	296±11

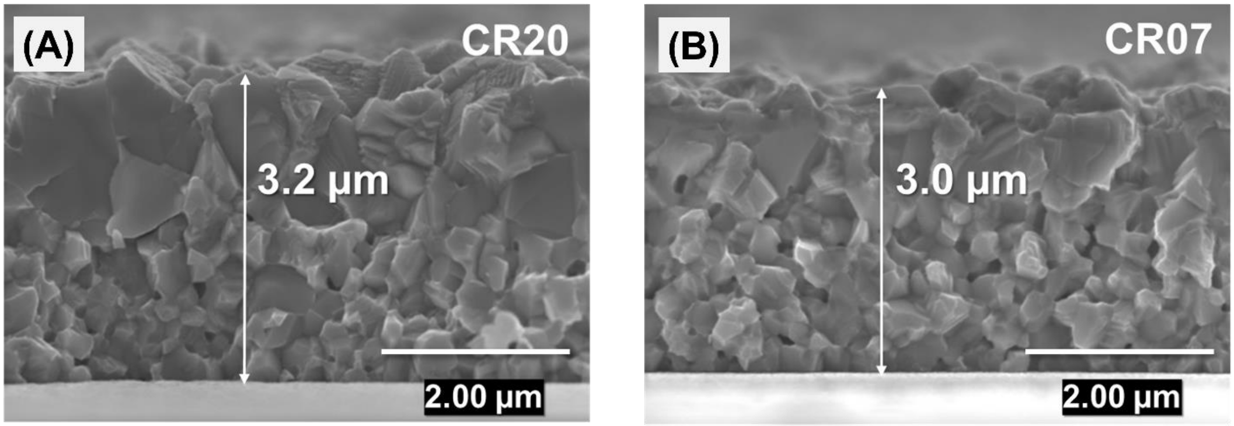

Figure S6. *Micrographs of cross-sectional scanning electron microscopy of the Cu(In,Ga)S_2 films processed with Cu-excess of A) 20% (CR20) and B) 7% (CR07).*

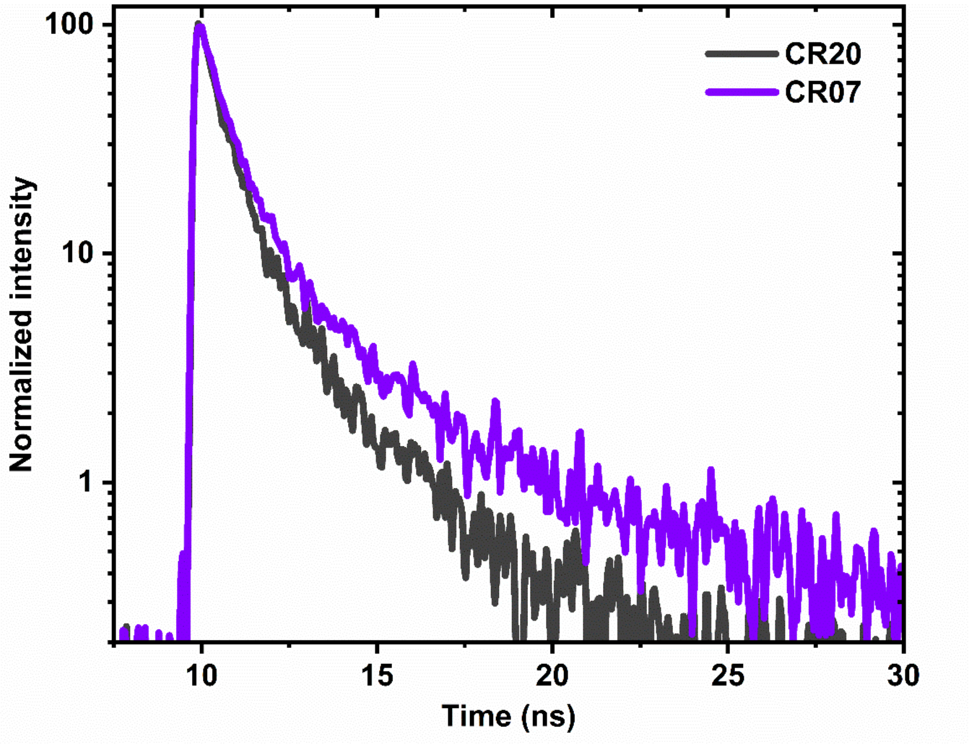

Figure S7. *Transient photoluminescence decay measured for CR20 and CR07.*

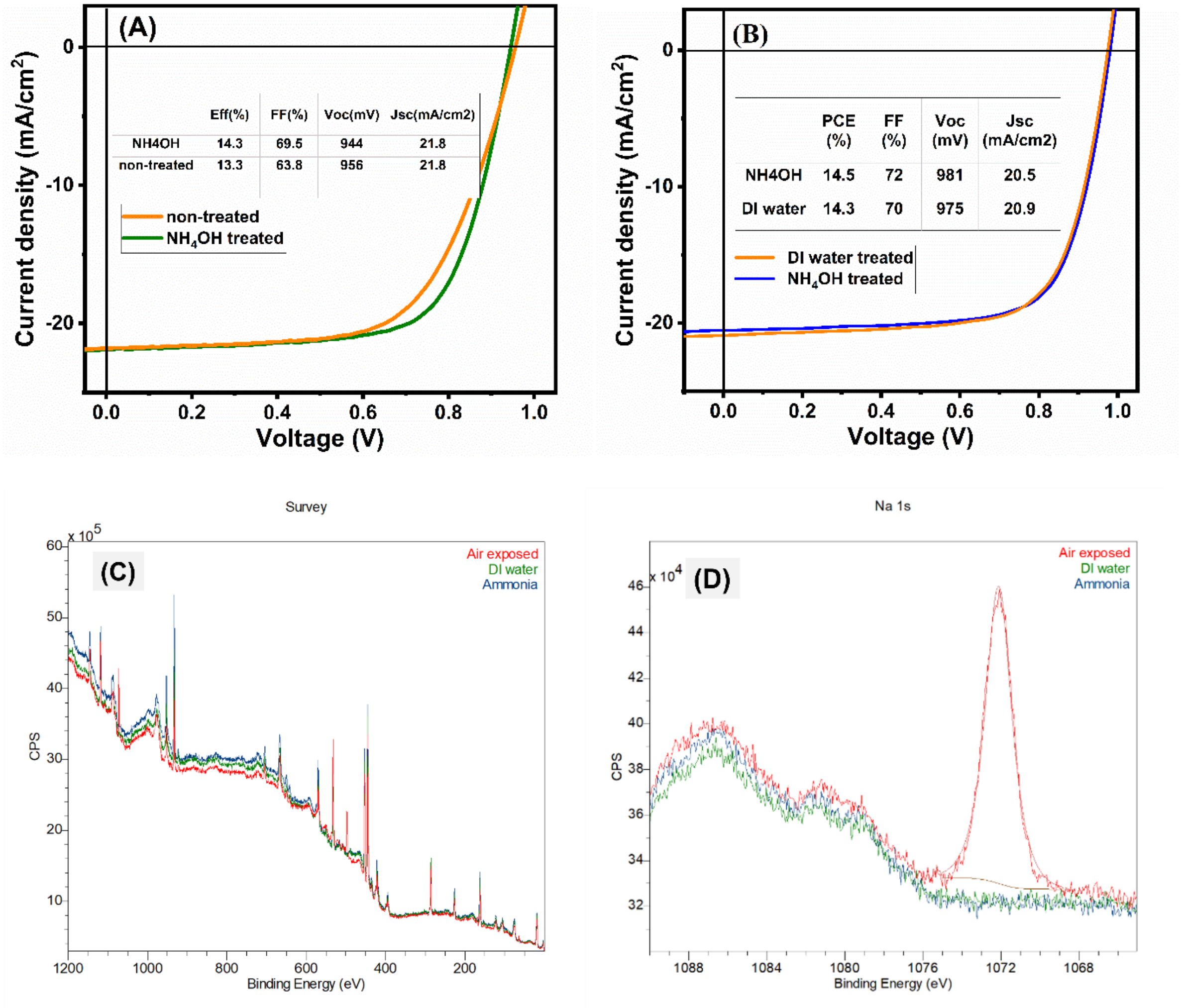

Figure S8. *Comparison of current-voltage characteristics of Cu(In,Ga)S_2 devices among A) non-treated and NH_4OH treatment, and B) among deionized (DI) water and NH_4OH treated absorbers. X-ray Photoelectron Spectroscopy survey spectra C) and Na 1s spectra D) of Cu(In,Ga)S_2 absorber air exposed in red, DI water treated in green and aqueous ammonia treated in blue rich.*

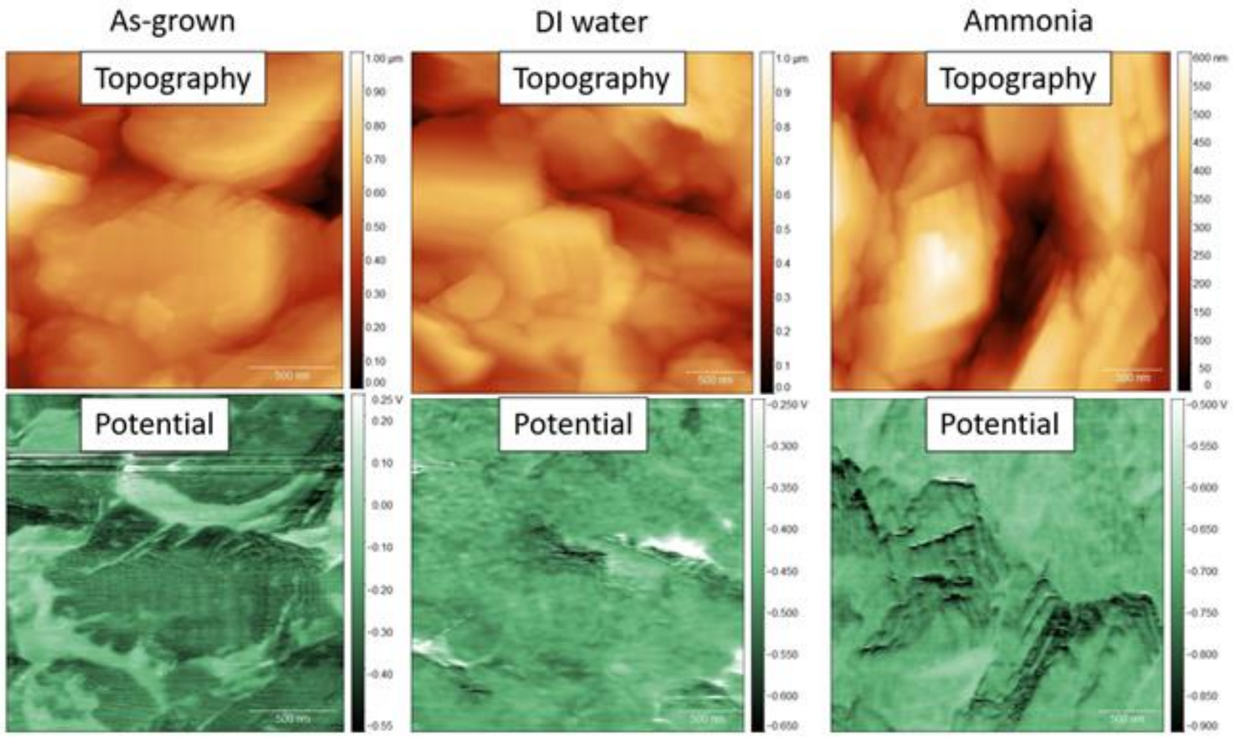

Figure S9. *Kelvin Probe Force Microscopy maps showing the topography and work-function on the surface of Cu(In,Ga)S_2 film as-grown, rinsed with with DI water and Ammonia. Brighter areas in Kelvin Probe Force Microscopy indicate lower work function.*

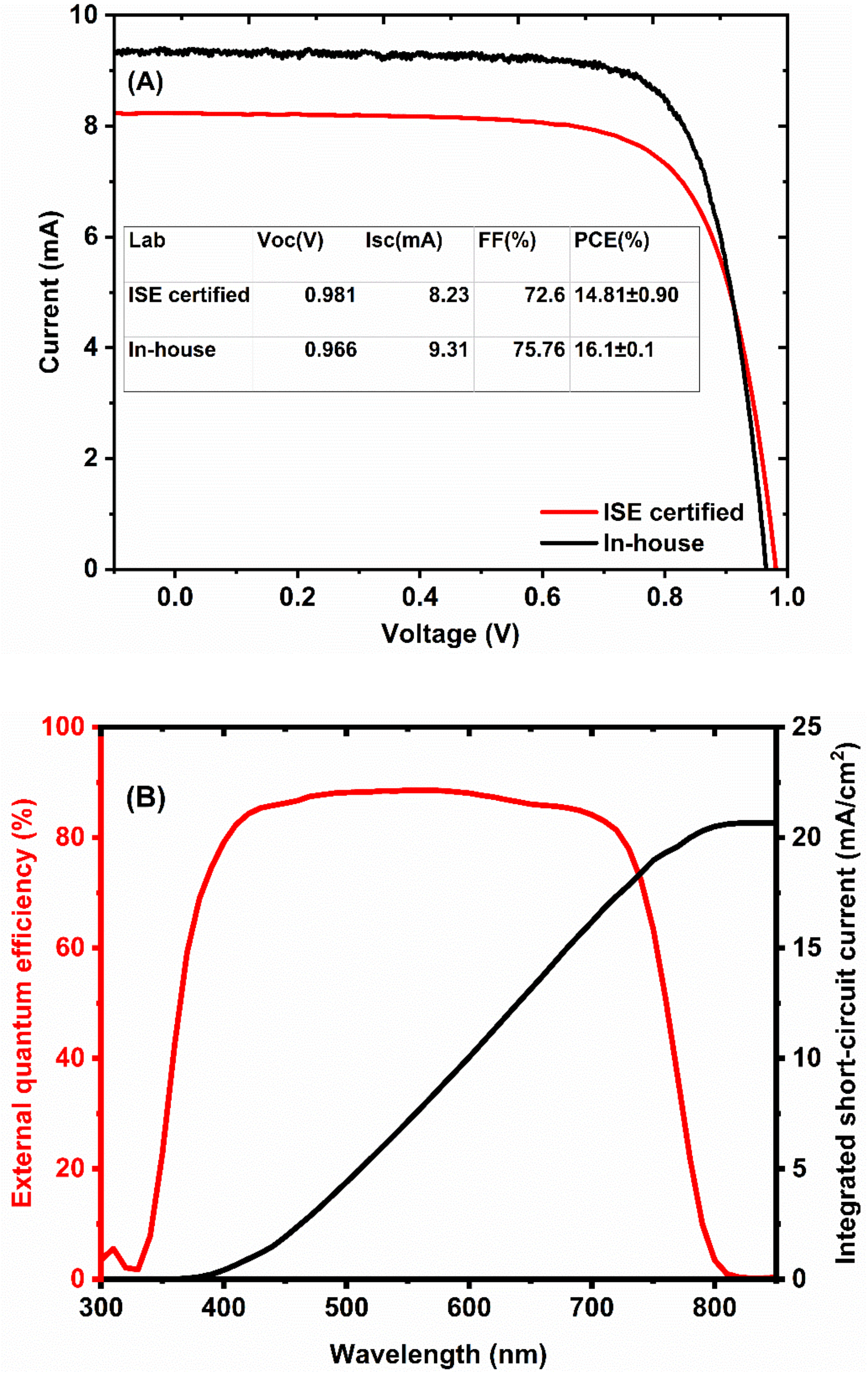

Figure S10. *A) Current–voltage characteristics for the champion solar cell obtained from in-house measurements and independently certified by Fraunhofer ISE. B) External quantum efficient curve and integrated short circuit current density obtained from the external quantum efficient curve of the champion solar cell device with an active area of 0.41 cm^2.*

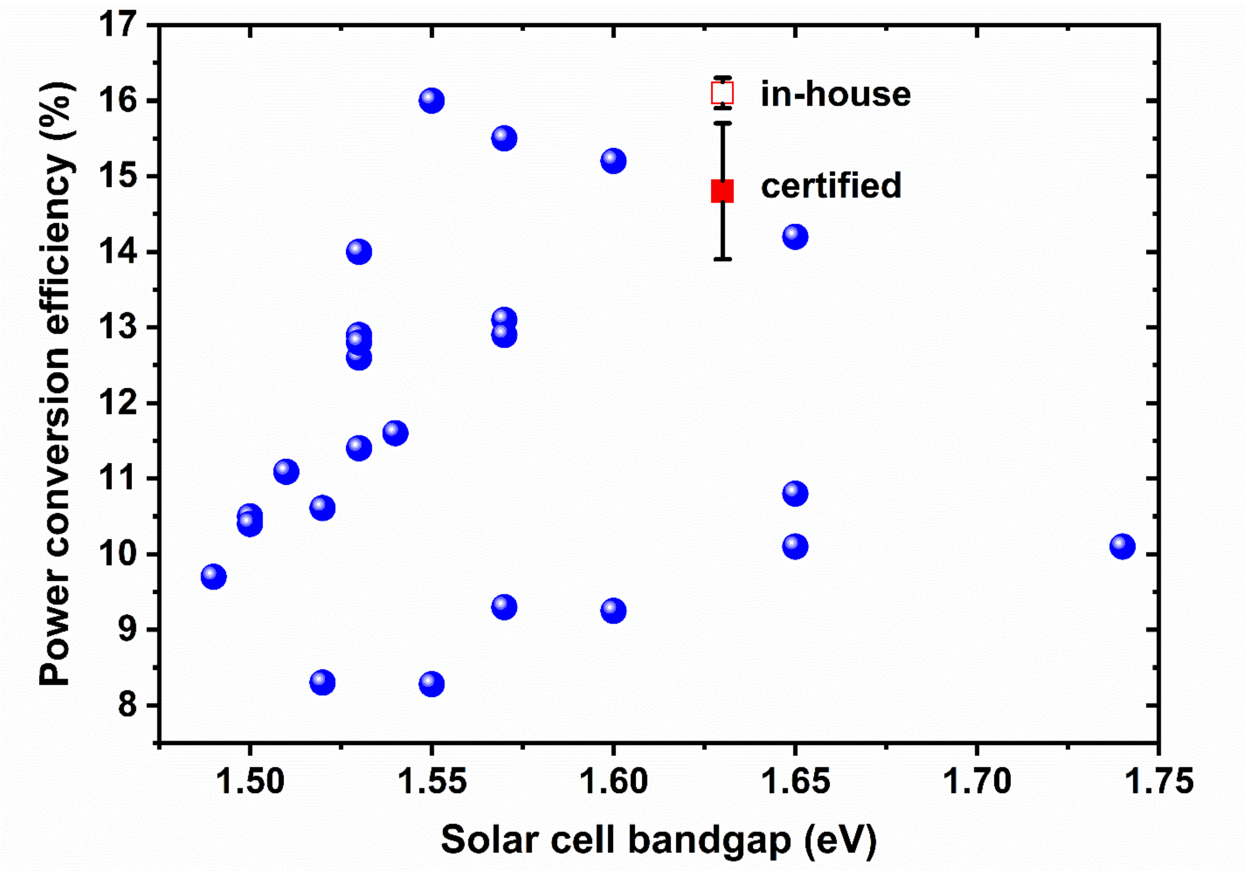

Figure S11. *Reported efficiency of different Cu(In,Ga)S_2 solar cells plotted against the respective bandgap determined from external quantum efficiency curves of the solar cells.*[1-10] *The in-house measured power conversion efficiency of our champion solar cell is shown in the open square whereas the independently certified power conversion efficiency is shown in the filled square.*

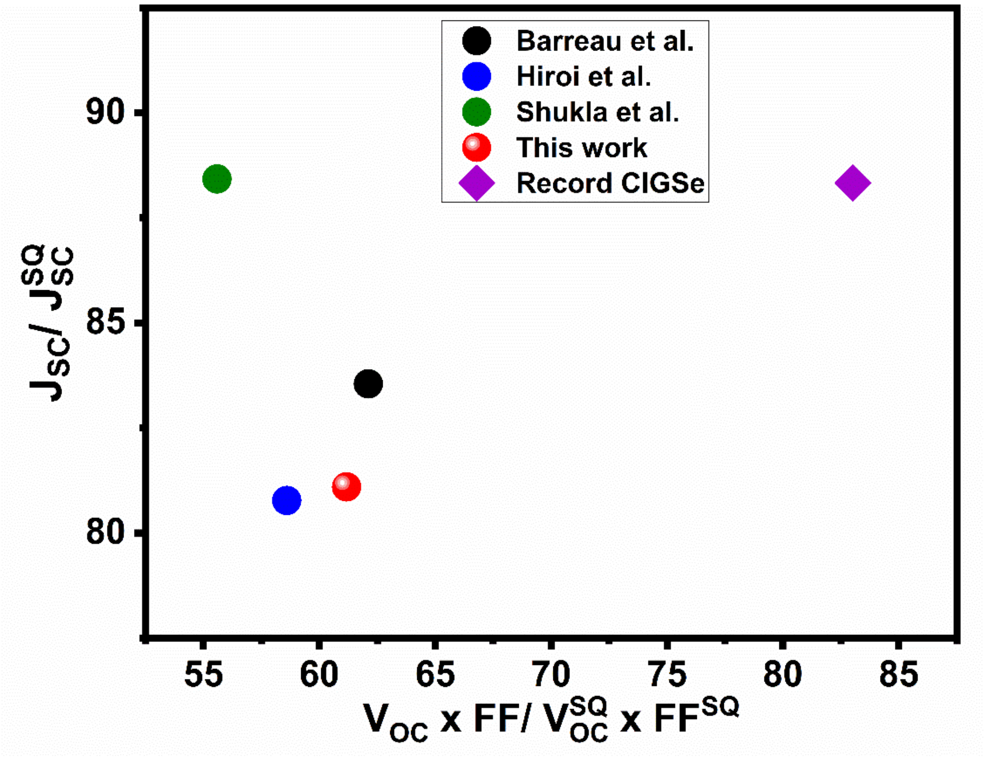

Figure S12. *Scatter plot showing the measured J_{SC} to J_{SC}^{SQ} and the product of measured V_{OC} and FF to V_{OC}^{SQ} and FF for Cu(In,Ga)S_2 devices with reported efficiency above 15%, and for reference the best selenide (Ag,Cu)(In,Ga)Se_2 device is also plotted in purple.*[1, 2, 4, 11]

Table S5. Optoelectronic characteristics of the champion device

First stage substrate temperature	Average CGI ratio	Average GGI ratio	Surface GGI ratio	E_g^{PL} (eV)	QFLS (meV)	$nrad_{loss}$ (meV)
435 °C	0.95	0.23	0.24	1.59	1056	260

References

[1] H. Hiroi, Y. Iwata, S. Adachi, H. Sugimoto, and A. Yamada, “New World-Record Efficiency for Pure-Sulfide Cu(In,Ga)S2 Thin-Film Solar Cell with Cd-Free Buffer Layer via KCN-Free Process,” *IEEE Journal of Photovoltaics,* vol. 6, pp. 760-763, 03/21, 2016.

[2] N. Barreau, E. Bertin, A. Crossay, O. Durand, L. Arzel, S. Harel, T. Lepetit, L. Assmann, E. Gautron, and D. Lincot, “Investigation of co-evaporated polycrystalline Cu (In, Ga) S2 thin film yielding 16.0% efficiency solar cell,” *EPJ Photovoltaics,* vol. 13, pp. 17, 2022.

[3] R. Kaigawa, A. Neisser, R. Klenk, and M.-C. Lux-Steiner, “Improved performance of thin film solar cells based on Cu (In, Ga) S2,” *Thin Solid Films,* vol. 415, no. 1-2, pp. 266-271, 2002.

[4] S. Shukla, M. Sood, D. Adeleye, S. Peedle, G. Kusch, D. Dahliah, M. Melchiorre, G.-M. Rignanese, G. Hautier, R. Oliver, and S. Siebentritt, “Over 15% efficient wide-band-gap Cu(In,Ga)S2 solar cell: Suppressing bulk and interface recombination through composition engineering,” *Joule*, 2021/06/07/, 2021.

[5] H. Hiroi, Y. Iwata, H. Sugimoto, and A. Yamada, “Progress Toward 1000-mV Open-Circuit Voltage on Chalcopyrite Solar Cells,” *IEEE Journal of Photovoltaics,* vol. 6, no. 6, pp. 1630-1634, 2016.

[6] S. Merdes, R. Mainz, J. Klaer, A. Meeder, H. Rodriguez-Alvarez, H. Schock, M. C. Lux-Steiner, and R. Klenk, “12.6% efficient CdS/Cu (In, Ga) S2-based solar cell with an open circuit voltage of 879 mV prepared by a rapid thermal process,” *Solar Energy Materials and Solar Cells,* vol. 95, no. 3, pp. 864-869, 2011.

[7] G. He, C. Yan, J. Li, X. Yuan, K. Sun, J. Huang, H. Sun, M. He, Y. Zhang, and J. A. Stride, “11.6% efficient pure sulfide Cu (In, Ga) S2 solar cell through a Cu-deficient and KCN-free process,” *ACS Applied Energy Materials,* vol. 3, no. 12, pp. 11974-11980, 2020.

[8] S. Merdes, R. Sáez-Araoz, A. Ennaoui, J. Klaer, M. C. Lux-Steiner, and R. Klenk, “Recombination mechanisms in highly efficient thin film Zn (S, O)/Cu (In, Ga) S 2 based solar cells,” *Applied Physics Letters,* vol. 95, no. 21, pp. 213502, 2009.

[9] T. Ohashi, Y. Hashimoto, and K. Ito, “Cu (In1-xGax) S2 thin-film solar cells with efficiency above 12%, fabricated by sulfurization,” *Japanese journal of applied physics,* vol. 38, no. 7A, pp. L748, 1999.

[10] H. Hiroi, Y. Iwata, K. Horiguchi, and H. Sugimoto, “960-mV open-circuit voltage chalcopyrite solar cell,” *IEEE Journal of Photovoltaics,* vol. 6, no. 1, pp. 309-312, 2015.

[11] J. Keller, K. Kiselman, O. Donzel-Gargand, N. M. Martin, M. Babucci, O. Lundberg, E. Wallin, L. Stolt, and M. Edoff, “High-concentration silver alloying and steep back-contact gallium grading enabling copper indium gallium selenide solar cell with 23.6% efficiency,” *Nature Energy*, pp. 1-12, 2024.
